# Supplementary material for: Constitutively Active SMAD2/3 Are Broad-Scope Potentiators of Transcription-Factor-Mediated Cellular Reprogramming
Source: Cell Stem Cell. 2017 Dec 7;21(6):791–805.e9. doi: 10.1016/j.stem.2017.10.013 (PMC5732323; doi:10.1016/j.stem.2017.10.013)
Supplement: Document S1. Figures S1–S6 [file mmc1.pdf]

Cell Stem Cell, Volume *21*

Supplemental Information

Constitutively Active SMAD2/3 Are Broad-Scope Potentiators of Transcription-Factor-Mediated Cellular Reprogramming

Tyson Ruetz, Ulrich Pfisterer, Bruno Di Stefano, James Ashmore, Meryam Beniazza, Tian V. Tian, Daniel F. Kaemena, Luca Tosti, Wenfang Tan, Jonathan R. Manning, Eleni Chantzoura, Daniella Rylander Ottosson, Samuel Collombet, Anna Johnsson, Erez Cohen, Kosuke Yusa, Sten Linnarsson, Thomas Graf, Malin Parmar, and Keisuke Kaji

Supplemental Figures and Legends

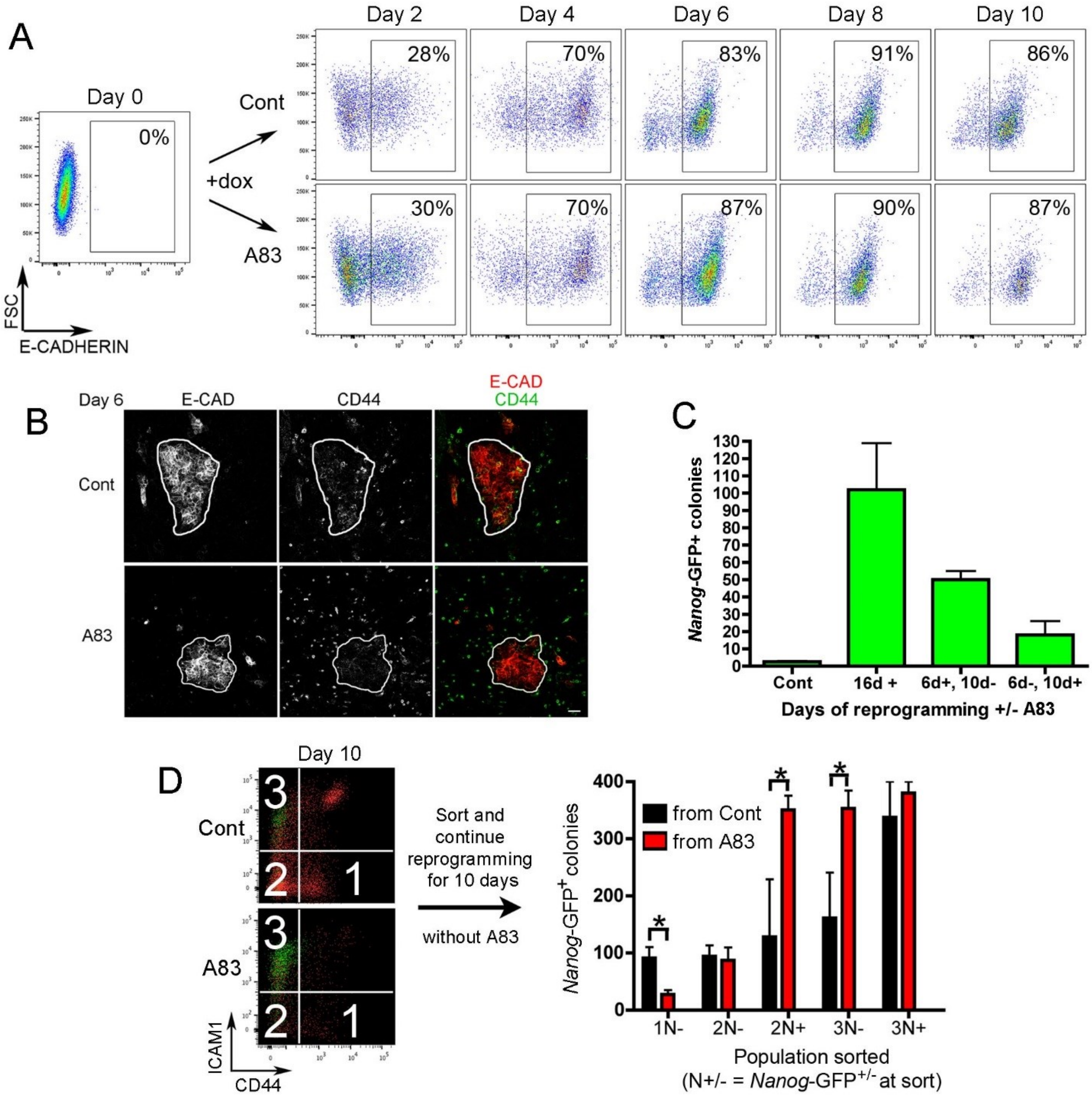

Figure S1, Related to Figure 1. Effects of the TGF-β inhibitor on reprogramming. (A) E-CADHERIN expression changes during reprogramming without (top panels) and with A83 (bottom panels). (B) Confocal microscopy images of E-CAD and CD44 expression at day 6 of reprogramming without (top panels) and with A83 (bottom panels). Scale bar is 50 μM. (C) Numbers of *Nanog*-GFP+ colonies in the absence (Cont) or presence of A83 for 16 days (16d+), only the first 6 days (6d+, 10d-), and the last 10 days (6d-, 10d+). These experiments were performed in the absence of VitC. (D) *Nanog*-GFP$^-$ or GFP$^+$ cells in gate 1, 2, 3 on day 10 of reprogramming without (top) or with A83 (bottom) were sorted. Ten days after continuous reprogramming in the absence of A83, numbers of *Nanog*-GFP$^+$ colonies were counted. Graphs represent averages of 3 independent experiments with 2 technical replicates. Error bars indicate standard deviation (s.d.). *P-value <0.05 based on a one-sided *t*-test.

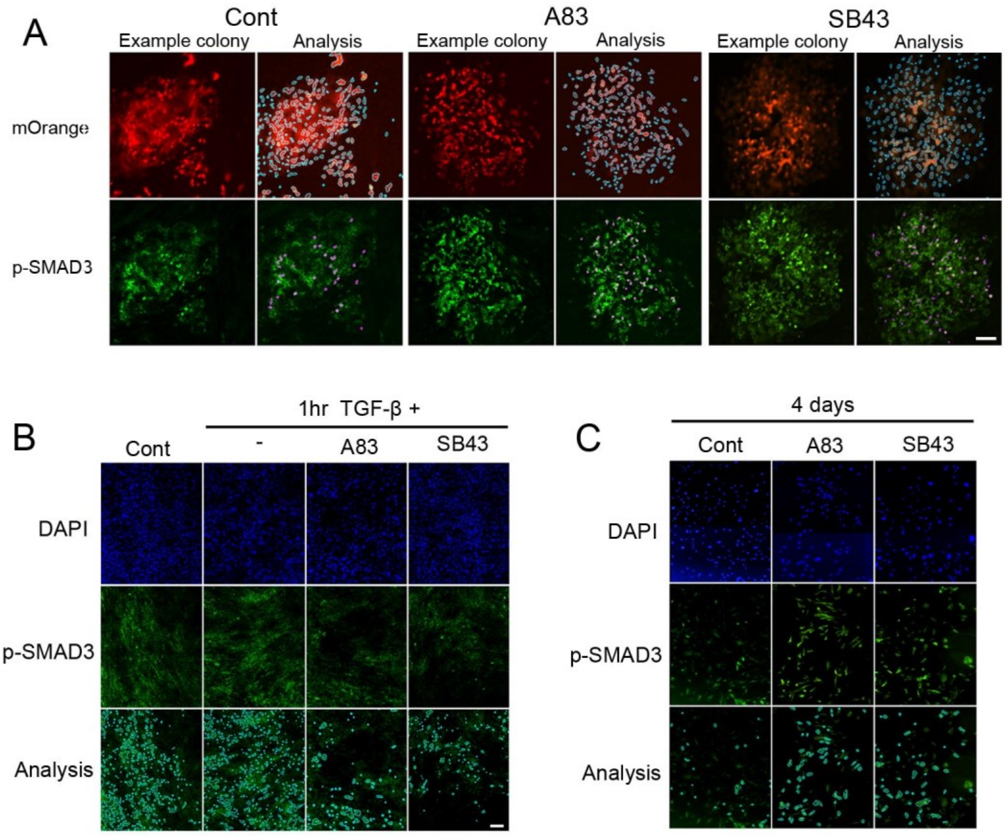

Figure S2, Related to Figure 2. Effects of the TGF-β and SMAD3 inhibitors on p-SMAD3 levels. (A) A close up of whole well images with Celigo imaging cytometer. Cells at day 4 of reprogramming without inhibitors (left), with A83 (middle) or SIS3 (right) were stained for p-SMAD3. Numbers of mOrange^{+} cells with strong p-SMAD3 foci in each well were quantified with segmentations as indicated. Scale bar is 100 µM. (B, C) Close up images of wild type MEFs cultured for 1 hour (B) or 4 days (C) without inhibitors (Cont, -), with A83 or SB43, stained and quantified for p-SMAD3 as shown. The 1 hour treatments groups were serum starved for 1 hour prior to treatment in ES media. Scale bars are 100 µM.

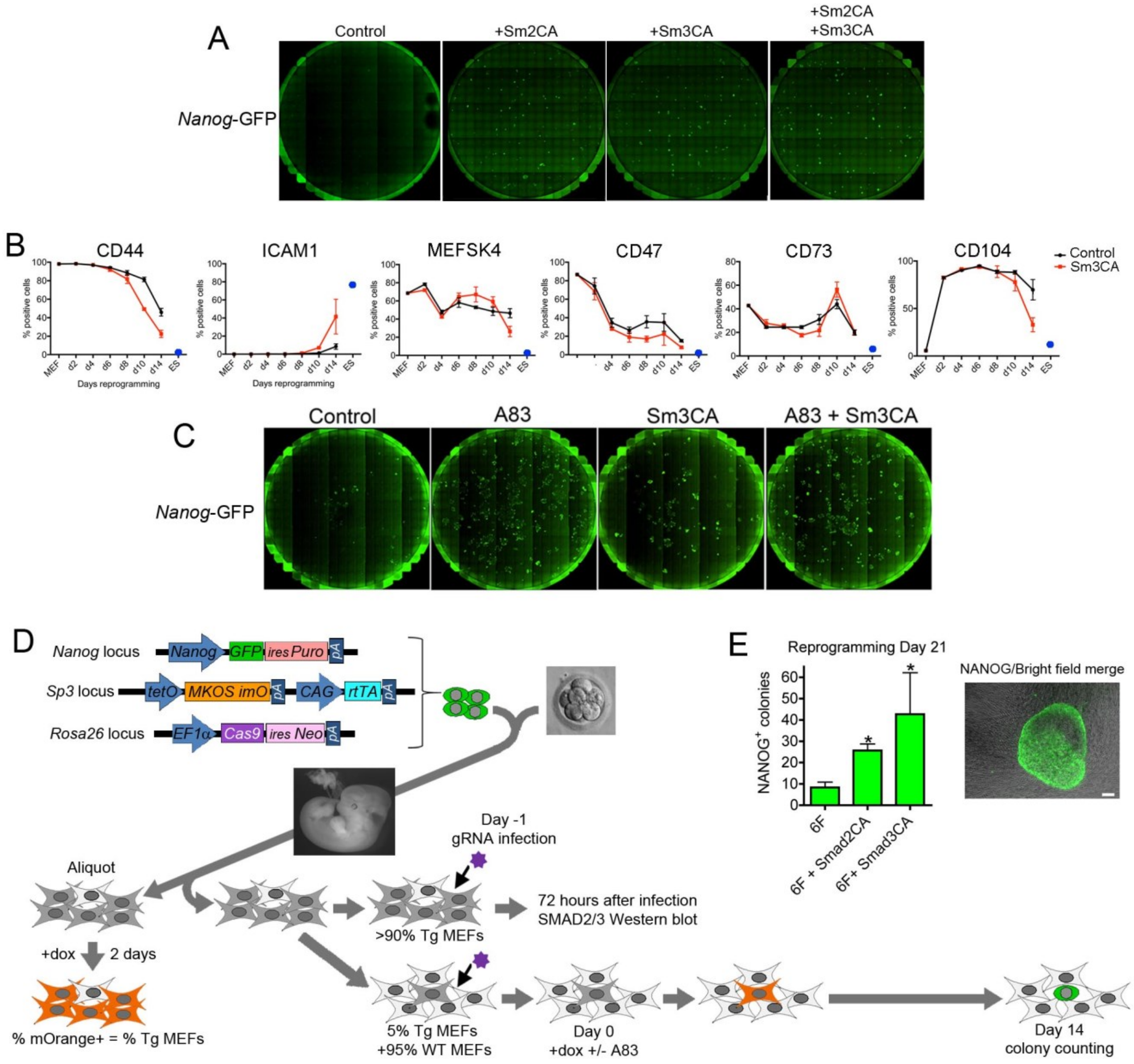

Figure S3, Related to Figure 3. Mouse and human cell reprogramming with Smad2/3CA. (A) Whole well images of reprogramming with control, *Smad2CA*, *Smad3CA*, or *Smad2CA* plus *Smad3CA* expression vector on day 15. Scale bar is 1 cm. (B) Flow cytometry analysis for CD44, ICAM1, MEFSK4, CD47, CD73 and CD104 during reprogramming in the presence (*Sm3CA*) and absence of (Control) of *Smad3CA* expression. (C) Whole well images of reprogramming with control, A83, *Smad3CA*, or A83 plus *Smad3CA* expression vector on day 15. (D) Reprogramming of *Smad2/3* double knockout MEFs. An ESC line with a Nanog-GFP reporter, doxycycline (dox)-inducible *MKOS-ires-mOrange*, CAG promoter-driven *rtTA*, EF1a promoter-driven *Cas9* was used to generate transgenic (Tg) MEFs. A portion of Tg MEFs were used to determine contribution of Tg cells by measuring the % of mOrange^{+} cells 2 days after dox administration. MEFs from each embryo with >90% Tg cell contribution were pooled and used for further experiments. Infection of gRNA expression viruses was performed onto >90% Tg MEFs and loss of SMAD2/3 protein was assessed 72 hours after infection. A mixture of Tg MEFs and wild-type (WT) MEFs (5 : 95) was infected with the same gRNA expression viruses in parallel and reprogrammed by administration of dox after 24 hours after infection in the presence or absence of A83. (E) Human dermal fibroblast reprogramming using episomal vectors with 6 factors (6F; *OCT3/4*, *SOX2*, *KLF4*, *L-MYC*, *LIN28*, shRNA against P53) with *Smad2CA* or *Smad3CA*. NANOG^{+} colony numbers were evaluated on day 21 of reprogramming. Graph represents averages of 3 independent experiments, with 2 technical replicates. Error bars indicate standard deviation (s.d.). *P-value <0.05 based on a one-sided *t*-test. Image shows a typical NANOG^{+} colony in 6F+*Smad3CA* reprogramming. Scale bar is 100 μM.

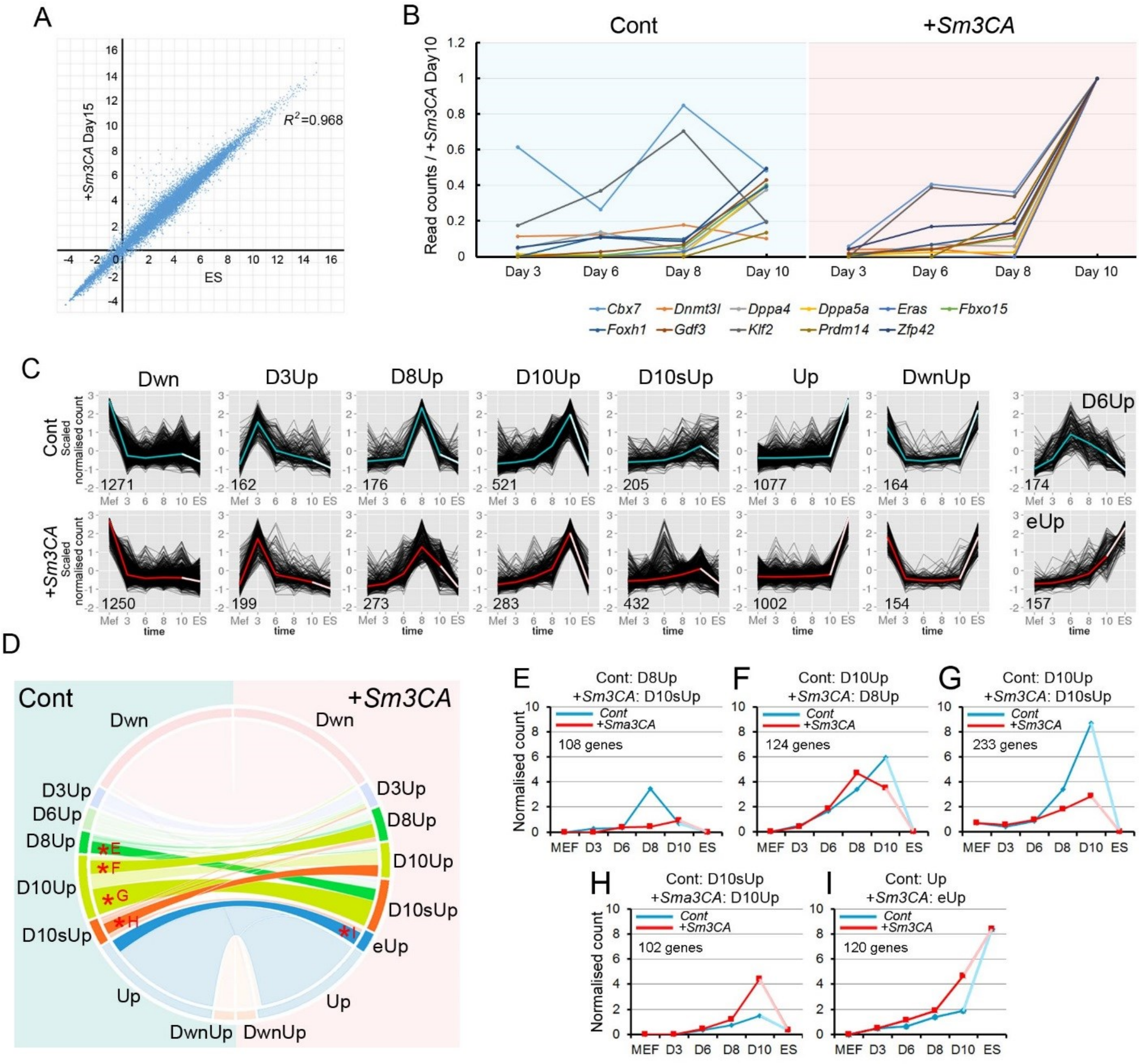

J

	Fold Enrichment Rank	GO biological process complete	REFLIST (22320)	Input (98)	Expected	Fold Enrichment	P-value
E<br>Cont: D8Up<br>+Sm3CA: D10sUp	1	oligodendrocyte differentiation (GO:0048709)	57	5	0.25	19.98	4.69E-02
	2	synapse organization (GO:0050808)	136	7	0.6	11.72	2.06E-02
	3	regulation of synaptic plasticity (GO:0048167)	160	8	0.7	11.39	4.80E-03
	4	regulation of neurotransmitter levels (GO:0001505)	171	8	0.75	10.66	7.85E-03
	5	regulation of synapse structure or activity (GO:0050803)	263	10	1.15	8.66	2.18E-03
	6	modulation of synaptic transmission (GO:0050804)	334	11	1.47	7.5	2.21E-03
	7	synaptic transmission (GO:0007268)	316	10	1.39	7.21	1.13E-02
	8	trans-synaptic signaling (GO:0099537)	316	10	1.39	7.21	1.13E-02
	9	anterograde trans-synaptic signaling (GO:0098916)	316	10	1.39	7.21	1.13E-02
	10	synaptic signaling (GO:0099536)	321	10	1.41	7.1	1.30E-02
F<br>Cont: D10Up<br>Sm3CA: D8Up	1	inflammatory response (GO:0006954)	387	11	1.94	5.66	3.47E-02
	2	innate immune response (GO:0045087)	472	12	2.37	5.07	3.86E-02
	3	immune response (GO:0006955)	861	19	4.32	4.4	4.74E-04
	4	immune system process (GO:0002376)	1646	24	8.26	2.91	1.45E-02
G<br>Cont: D10Up<br>Sm3CA: D10sUp	1	cellular response to interferon-beta (GO:0035458)	30	12	0.27	44.64	1.30E-12
	2	response to interferon-beta (GO:0035456)	36	14	0.32	43.4	5.57E-15
	3	T cell migration (GO:0072678)	11	4	0.1	40.58	2.74E-02
	4	response to interferon-alpha (GO:0035455)	19	5	0.17	29.37	7.65E-03
	5	negative regulation of viral genome replication (GO:0045071)	40	10	0.36	27.9	4.37E-08
	6	defense response to virus (GO:0051607)	127	23	1.14	20.21	5.98E-19
	7	response to interferon-gamma (GO:0034341)	72	13	0.65	20.15	1.60E-09
	8	cellular response to interferon-gamma (GO:0071346)	52	9	0.47	19.32	1.24E-05
	9	regulation of viral genome replication (GO:0045069)	65	10	0.58	17.17	4.62E-06
	10	response to virus (GO:0009615)	166	25	1.49	16.81	6.10E-19

Figure S4, Related to Figure 4. Accelerated gene expression changes toward a pluripotent state by *Smad3CA*. (A) Global gene expression comparison between +*Sm3CA* reprogramming *Nanog*-GFP$^+$ cells on day 15 and ESCs. (B) Pluripotency gene expression changes during reprogramming with control (Cont) or *Smad3CA* (+*Sm3CA*) vector expression. Among 30 pluripotency genes highlighted in Figure 3A, 11 genes with >2-fold expression in the +*Sm3CA* sample compared to the control sample on day 10 are shown. Mean read counts of triplicates were normalized by the values of the +*Sm3CA* Day 10 sample. (C) Scaled expression profiles for genes clustered by similar expression patterns during reprogramming with control (Cont) and *Smad3CA* (+*Sm3CA*) vector expression. The blue and red lines represent mean values of each cluster. Numbers of genes in each category are indicated in each panel. (D) Distribution of genes classified in each category in control (left) and +*Smad3CA* (right) reprogramming. Asterisks indicate cross-classified gene groups with more than 100 genes. (E-I) Un-scaled expression patterns of the cross-classified gene groups *E-I in D. The median values of normalized counts in each group are indicated as blue or red lines from control or +*Smad3CA* reprogramming, respectively. (J) Gene ontology enriched in *E-G groups with PANTHER (Thomas et al., 2003). No enrichment was observed in *H-I groups.

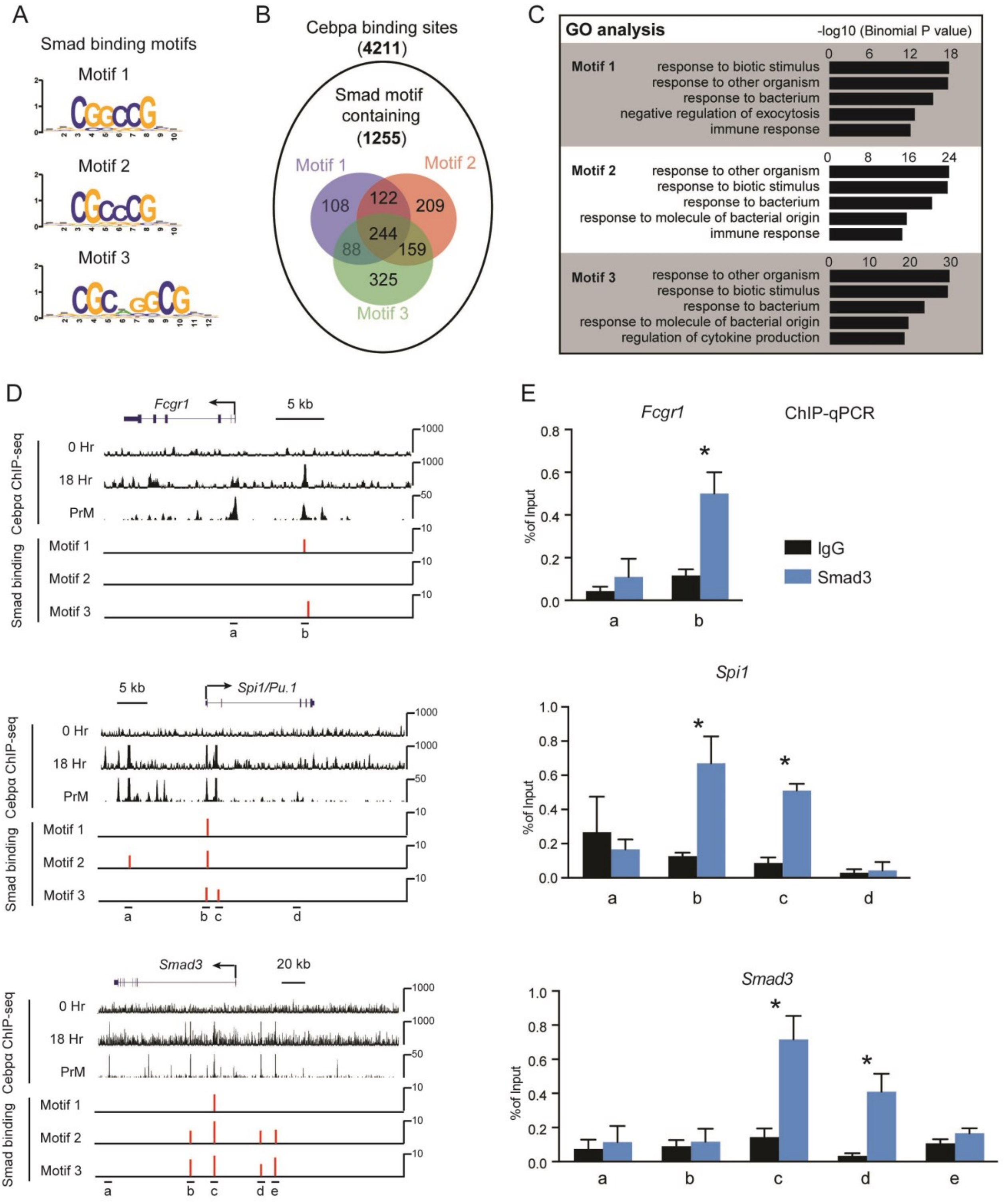

Figure S5, Related to Figure 6. Smad3 binds to Cebpα-bound genomic loci during transdifferentiation from B cells to macrophages. (A) Three Smad-binding motifs found by motif discovery in the vicinity of 4211 Cebpα peak summits. The Cebpα ChIP-seq was performed 18 hours after its induction in primary mouse CD19+ B cells. (GSE71218) (Di Stefano et al., 2016) (B) Venn diagram showing numbers of the three motifs from (A) and the overlap between them. (C) Gene Ontology (GO) enrichments for Cebpα target genes associated with indicated Smad motif from (A). (D) Representative snapshots of loci showing binding of Cebpα (ChIP-seq data in black) and occurrences of Smad-binding motifs (red vertical lines, vertical axes represent the weight of the motif matrix at this position). Cebpα ChIP-seqs were performed in mouse CD19+ B cells (0 Hr) (GSE71218), 18 hours after the induction of Cebpα (18 Hrs) (GSE71218) and in mouse primary macrophages (PrM) (GSE53362) (van Oevelen et al., 2015). (E) Validation of Smad binding by ChIP-qPCR in C10 pre-B cell line induced for Cebpα expression for 18 hrs. S.D. are from two biological replicates (* $P<0.05$).

A

Intrinsic properties	Cont	+*Sm2CA*	+*Sm3CA*
Resting membrane potential (mV)	-30.14 ± 3.83	-27.11 ± 3.85	-38.20 ± 2.78
Capacitance (pF)	3.98 ± 0.55	7.62 ± 0.70*	8.20 ± 0.69*
Membrane resistance (GΩ)	4.24 ± 1.17	4.40 ± 0.47	4.62 ± 0.93
Inward Na+ current	73.14 ± 43.7	332 ± 46.99*	353.13 ± 52.37*
Outward K+ current	299.28 ± 59.78	665 ± 155.8	827.20 ± 90.04*

* P<0.05

B

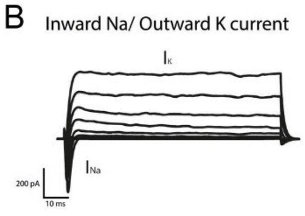

C

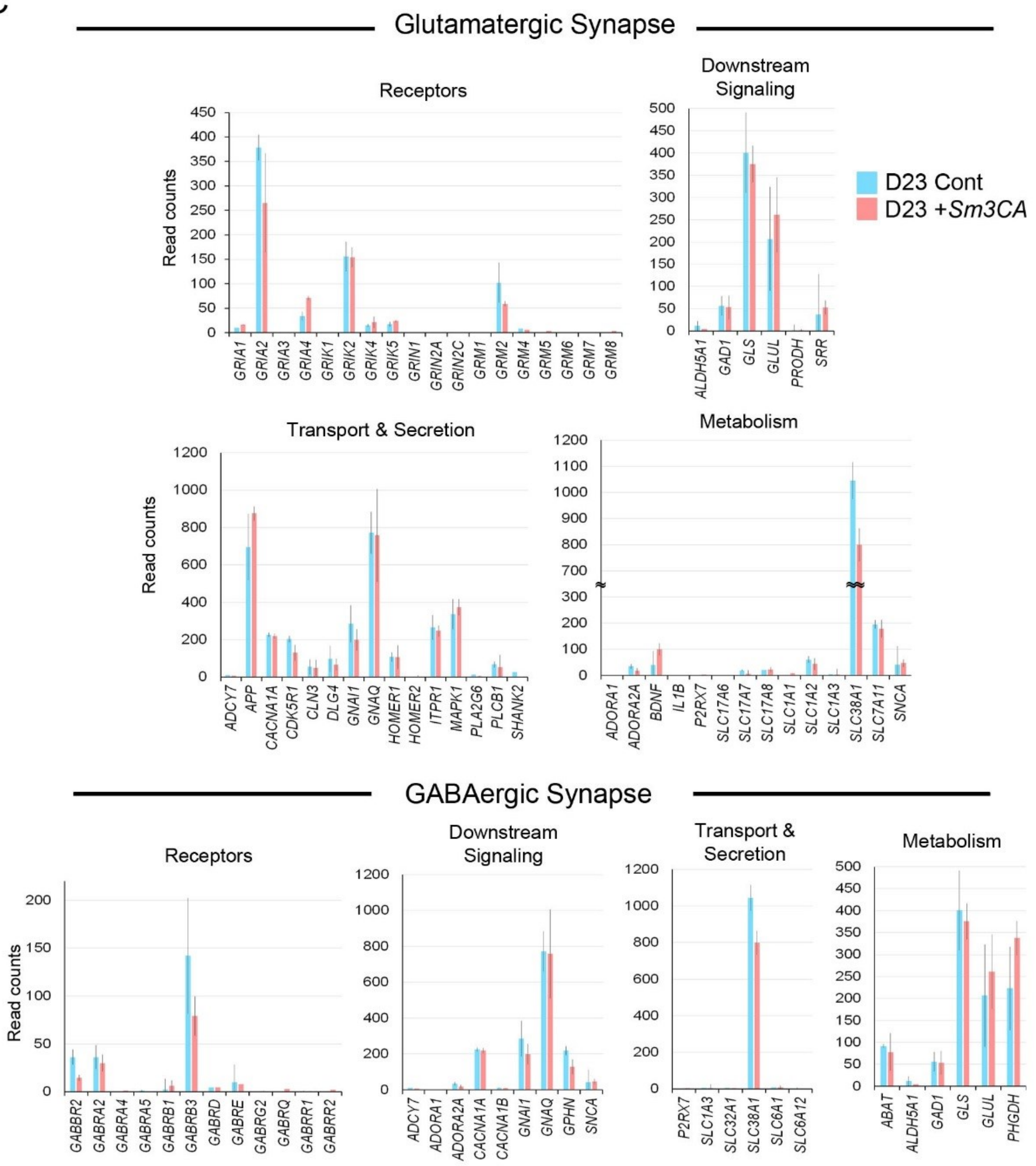

Figure S6, Related to Figure 7. Characters of iNs generated in the presence and absence of Smad3CA. (A) A summary of the main electrophysiological intrinsic membrane parameters measured for hiNs induced with *ABMN* (Cont) or *ABMN* plus *Sm2CA* or *Sm3CA* (25d after conversion). Both membrane capacitance and Na^+, K^+ currents were significantly greater (P<0.05 unpaired t-test) in +*Sm2/3CA* conditions compared to control, indicating a more mature neuronal phenotype. (B) Representative trace of whole-cell currents from hiNs induced with *ABMN* and *Sm3CA*. Trace shows inward sodium (Na^+) and outward potassium (K^+) currents upon depolarization indicative of functional expression of these membrane receptors and a neuronal and mature phenotype. 14 of 15 cells showed the recorded activity. (C) Expression of glutamatergic and GABAergic neuron associated genes. Day 23 *ABMN* (D23 Cont) iNs in pale blue and *ABMN*+*Smad3CA* (D23 +*Sm3CA*) iNs in pink. Mean read counts of triplicates from the RNA-seq are shown. Error bars indicate standard deviation.
