# Supplementary material for: Constitutively Active SMAD2/3 Are Broad-Scope Potentiators of Transcription-Factor-Mediated Cellular Reprogramming
Source: Cell Stem Cell. 2017 Dec 7;21(6):791–805.e9. doi: 10.1016/j.stem.2017.10.013 (PMC5732323; doi:10.1016/j.stem.2017.10.013)
Supplement: Document S2. Article plus Supplemental Information [file mmc5.pdf]

Article

Cell Stem Cell

Constitutively Active SMAD2/3 Are Broad-Scope Potentiators of Transcription-Factor-Mediated Cellular Reprogramming

Graphical Abstract

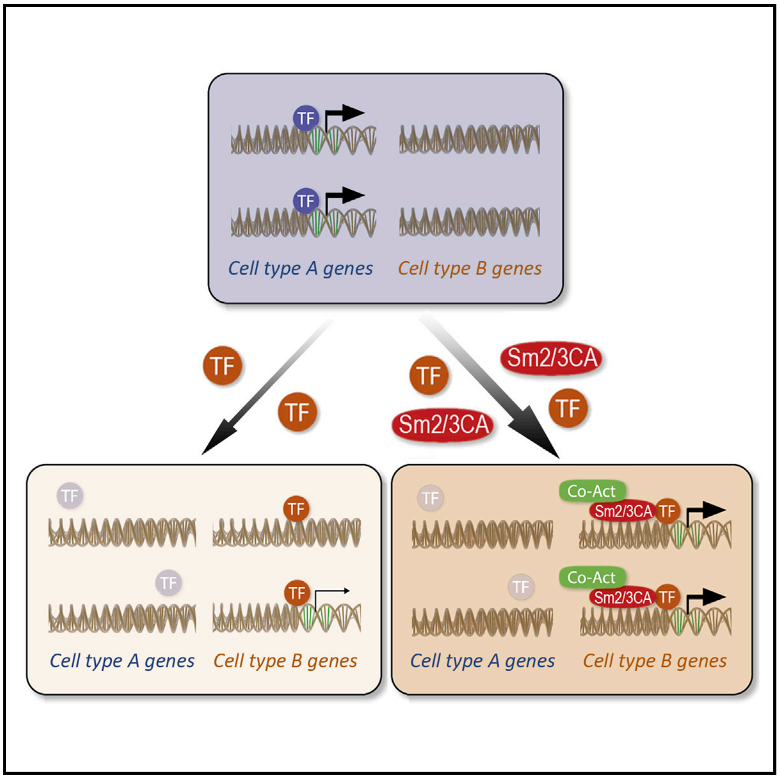

Highlights

- Enhancement of iPSC reprogramming by TGF-βR inhibition is independent of SMAD2/3
- Constitutively active SMAD2/3 improves reprogramming efficiency and kinetics
- SMAD3 co-occupies OCT4 targets during reprogramming
- Constitutively active SMAD2/3 also boosts TF-mediated direct lineage reprogramming

Authors

Tyson Ruetz, Ulrich Pfisterer, Bruno Di Stefano, ..., Thomas Graf, Malin Parmar, Keisuke Kaji

Correspondence

keisuke.kaji@ed.ac.uk

In Brief

Ruetz et al. show that constitutively active SMAD2/3 has a surprising ability to boost the efficiency of cell reprogramming both to iPSCs and across lineages and may therefore be a general factor that can enhance transcription-factor-mediated reprogramming in a variety of contexts.

Ruetz et al., 2017, Cell Stem Cell *21*, 791–805
December 7, 2017 © 2017 The Authors. Published by Elsevier Inc.
https://doi.org/10.1016/j.stem.2017.10.013

Cell Stem Cell
Article

Constitutively Active SMAD2/3 Are Broad-Scope Potentiators of Transcription-Factor-Mediated Cellular Reprogramming

Tyson Ruetz,[1] Ulrich Pfisterer,[2] Bruno Di Stefano,[3,4,9,10] James Ashmore,[1] Meryam Beniazza,[1] Tian V. Tian,[3,4] Daniel F. Kaemena,[1] Luca Tosti,[1] Wenfang Tan,[1] Jonathan R. Manning,[1] Eleni Chantzoura,[1] Daniella Rylander Ottosson,[2] Samuel Collombet,[5] Anna Johnsson,[6] Erez Cohen,[1] Kosuke Yusa,[7] Sten Linnarsson,[6] Thomas Graf,[3,4,8] Malin Parmar,[2] and Keisuke Kaji[1,11,*]

[1]MRC Centre for Regenerative Medicine, University of Edinburgh, Edinburgh BioQuarter, 5 Little France Drive, Edinburgh EH16 4UU, Scotland, UK
[2]Department of Experimental Medical Science, Developmental and Regenerative Neurobiology, Wallenberg Neuroscience Center and Lund Stem Cell Center, Lund University, 22184 Lund, Sweden
[3]Centre for Genomic Regulation, Dr Aiguader 88, 08003 Barcelona, Spain
[4]Universitat Pompeu Fabra (UPF), Dr Aiguader 88, 08003 Barcelona, Spain
[5]Institut de Biologie de l'Ecole Normale Supérieure, CNRS UMR8197, INSERM U1024, 75005 Paris, France
[6]Laboratory for Molecular Neurobiology, Department of Medical Biochemistry and Biophysics, Karolinska Institute, Scheeles väg 1, SE-171 77 Stockholm, Sweden
[7]Wellcome Trust Sanger Institute, Hinxton, Cambridge CB10 1SA, UK
[8]The Barcelona Institute of Science and Technology, Carrer del Comte d'Urgell 187, Building 12 (BIST), 08036 Barcelona, Spain
[9]Present address: Department of Molecular Biology, Massachusetts General Hospital, 185 Cambridge Street, Boston, MA 02114, USA
[10]Present address: Department of Stem Cell and Regenerative Biology, Harvard University, Cambridge, MA 02138, USA
[11]Lead Contact
*Correspondence: keisuke.kaji@ed.ac.uk
https://doi.org/10.1016/j.stem.2017.10.013

SUMMARY

Reprogramming of cellular identity using exogenous expression of transcription factors (TFs) is a powerful and exciting tool for tissue engineering, disease modeling, and regenerative medicine. However, generation of desired cell types using this approach is often plagued by inefficiency, slow conversion, and an inability to produce mature functional cells. Here, we show that expression of constitutively active SMAD2/3 significantly improves the efficiency of induced pluripotent stem cell (iPSC) generation by the Yamanaka factors. Mechanistically, SMAD3 interacts with reprogramming factors and co-activators and co-occupies OCT4 target loci during reprogramming. Unexpectedly, active SMAD2/3 also markedly enhances three other TF-mediated direct reprogramming conversions, from B cells to macrophages, myoblasts to adipocytes, and human fibroblasts to neurons, highlighting broad and general roles for SMAD2/3 as cell-reprogramming potentiators. Our results suggest that co-expression of active SMAD2/3 could enhance multiple types of TF-based cell identity conversion and therefore be a powerful tool for cellular engineering.

INTRODUCTION

Cell-type-specific transcription factors (TFs), which are responsible for specifying unique cellular identities, are often described as master TFs. Those master TFs not only play critical roles in self-renewal or cell fate specification during normal development but also can be used to drive cell identity conversions *in vitro* and *in vivo* (Graf, 2011). The first demonstration of cell identity conversion by an exogenous master TF was in 1987, with overexpression of *MyoD* in fibroblasts resulting in the generation of myoblasts (Davis et al., 1987). Follow-up studies accomplished TF-mediated transdifferentiation of hematopoietic lineages (Kulessa et al., 1995; Xie et al., 2004), which led to Takahashi and Yamanaka (2006) demonstrating the power of this strategy by generating induced pluripotent stem cells (iPSCs) from differentiated cells with only four TFs (*Oct4*, *Sox2*, *Klf4*, and *c-Myc*). Inspired by these seminal works, several different cell types have been generated by master-TF-mediated cellular identity conversions (Bussmann et al., 2009; Feng et al., 2008; Kajimura et al., 2009; Pfisterer et al., 2011; Vierbuchen et al., 2010), holding tremendous promise for cellular engineering and disease modeling. While overexpressing cell-type-specific master TFs is a key concept in forced cell identity conversions, the set of TFs, culture conditions, functionality of the resulting cells, and duration of conversion largely vary. Whether common molecular mechanisms are able to potentiate cell fate conversions across different models has remained to be explored. It has recently been demonstrated that CAF-1 is a common roadblock in three different master-TF-mediated cell conversions (Cheloufi et al., 2015). Further investigation of reprogramming mechanisms could unveil common molecular machineries that might similarly be involved in various cell conversion models, ultimately allowing for more efficient and faithful forced cell identity changes.

Among the several TF-mediated cell conversions, the generation of iPSCs is one of the most extensively studied systems. Several small molecules, targeting various epigenetic or signaling pathways, have been identified to enhance the reprogramming

This is an open access article under the CC BY license (http://creativecommons.org/licenses/by/4.0/).

process and even replace one or more of the Yamanaka factors, including transforming growth factor β receptor (TGF-βR) inhibitors (Ichida et al., 2009; Li et al., 2010; Maherali and Hochedlinger, 2009). TGF-β signaling counteracts the mesenchymal to epithelial transition (MET) (Xu et al., 2008), an essential early event for fibroblasts to become iPSCs (Li et al., 2010; Redmer et al., 2011; Samavarchi-Tehrani et al., 2010), providing a possible mechanism for how inhibition of this pathway promotes iPSC generation. However, TGF-βR inhibitors also improve reprogramming efficiency of epithelial cells (Li et al., 2010) and when added at post-MET stages of the reprogramming process (Ichida et al., 2009; Li et al., 2010), suggesting additional mechanisms of the reprogramming enhancement exist.

TGF-βRs transmit signals intracellularly through SMAD2/3-dependent and independent pathways (Derynck and Zhang, 2003). When phosphorylated by the activated TGF-βRs, SMAD2/3 translocate to the nucleus with SMAD4 and regulate transcription of hundreds of genes in a highly context-dependent manner (Massagué, 2012). SMAD2/3 interact with a range of TFs, as well as transcriptional activators, silencers, and nucleosome modifiers, including CBP/p300 transcriptional regulators (Feng et al., 1998), switch/sucrose non-fermentable (SWI/SNF) chromatin remodelers (Xi et al., 2008), the mixed lineage leukemia (MLL) histone H3 lysine 4 (H3K4) methyltransferase complex (Bertero et al., 2015), and the Mediator complex (Kato et al., 2002). Because SMAD2/3 have weak binding affinity to DNA (Shi et al., 1998), their robust interaction with the genome relies on other TFs, which can partially explain cell-type and context-dependent cellular responses to TGF-β activation (Derynck and Zhang, 2003; Ikushima and Miyazono, 2010; Massagué et al., 2005). Recent genome-wide chromatin immunoprecipitation followed by sequencing (ChIP-seq) analyses demonstrated that SMAD3 binds almost entirely unique target loci across the genome in a given cell type, co-localizing with OCT4, SOX2, and NANOG in embryonic stem cells (ESCs), PU.1 in pro-B cells, and MYOD1 in myotubes (Mullen et al., 2011). Thus, SMAD2/3 are powerful gene expression regulators, yet their potential positive role in iPSC generation has been disregarded, because the inhibition of the upstream molecule, TGF-βR, enhances reprogramming efficiency, and because their involvement in other master-TF-mediated cell identity conversions has not been explored.

Here, we demonstrate that TGF-βR inhibitor treatment circumvents reprogramming-dependent upregulation of p19ARF, a senescence inducer, independently of the promotion of the MET. Unexpectedly, we found that active SMAD2/3 increases when cells are cultured in the presence of TGF-βR inhibitors for prolonged periods. Following that observation, we assessed the impact of constitutively active SMAD2/3 (Smad2/3CA) on reprogramming and found a remarkable boost in efficiency and acceleration of the process. However, Cas9-mediated double knockout of *Smad2/3* revealed that endogenous SMAD2/3 was not responsible for TGF-βR-inhibitor-mediated reprogramming enhancement, suggesting that other receptor downstream targets are involved. Irrespectively, we discovered that overexpressed SMAD3CA physically interacted with reprogramming factors and localized at OCT4 target loci during reprogramming. Moreover, active SMAD3 could also enhance three other master-TF-mediated cell identity conversions. This work highlights SMAD2/3 as common powerful cofactors that potentiate diverse forced cell identity conversions with master TFs.

RESULTS

TGF-βR Inhibition Enhances Reprogramming Independently of the MET

To explore how TGF-βR inhibitors enhance reprogramming (Ichida et al., 2009; Li et al., 2010; Maherali and Hochedlinger, 2009), we first confirmed the beneficial effect of the ALK4/5/7 inhibitor A83-01 (A83) (Tojo et al., 2005) using mouse embryonic fibroblasts (MEFs) with doxycycline (dox)-inducible Yamanaka factors (*c-Myc*, *Klf4*, *Oct4*, and *Sox2*) and an mOrange reporter of transgene expression (MKOS-ires-mOrange) (Chantzoura et al., 2015). The addition of A83 to reprogramming media resulted in a 3-fold increase in reprogramming efficiency, measured by the number of colonies positive for *Nanog*-GFP reporter (Figure 1A). TGF-βR inhibitors are thought to facilitate reprogramming, at least in part, by enhancing the transition to an epithelial-like state (Li et al., 2010). However, over 70% of cells demonstrated E-CADHERIN expression after day 4 of reprogramming either in the presence or absence of A83 in this reprogramming system with a polycistronic reprogramming vector containing full-length *Klf4*, as previously reported (Figures 1B, S1A, and S1B) (Chantzoura et al., 2015; Kim et al., 2015; O'Malley et al., 2013). These results suggest that TGF-βR inhibition facilitates reprogramming independently of the acquisition of epithelial character in this system. During reprogramming, MKOS-expressing transgenic (Tg; mOrange$^+$) MEFs were found to proliferate more in the presence of A83, while the proliferation of surrounding wild-type MEFs was unaffected (Figure 1C). Real-time PCR and immunofluorescence revealed that transient upregulation of p19ARF, a p53-dependent cell-cycle arrest and apoptosis inducer, was suppressed in the Tg cells on day 4 of reprogramming in the presence of A83 (Figures 1D and 1E). Thus, one of the positive effects of A83 is anti-senescence/apoptosis at the early stage of reprogramming, which is consistent with the fact that A83 treatment only at the initial stages of reprogramming is also beneficial (Figure S1C) (Maherali and Hochedlinger, 2009). In addition, analysis of reprogramming progression with the cell-surface markers CD44 and ICAM1 indicated that the speed of reprogramming was also affected by the inhibitor (Figure 1F). MEFs with high CD44 and broad ICAM1 expression reach an iPSC state by going through gates 1, 2, and 3, which are marked by CD44 and ICAM1 expression levels (Figure 1F, day 0) (O'Malley et al., 2013). These CD44 and ICAM1 expression changes were accelerated in the presence of A83; for example, >50% of cells had already downregulated CD44 at day 8 as compared to ~15% of control cells (Figure 1F). Moreover, with the addition of A83, 33.7% of cells were *Nanog*-GFP$^+$ at day 8, in contrast to 1.6% in the absence of A83, indicating a robust acceleration of reprogramming. Late reprogramming intermediates generated in the presence of A83 were 2- to 2.5-fold more likely to form *Nanog*-GFP$^+$ colonies when flow-sorted based on CD44/ICAM1/*Nanog*-GFP expression profiles at day 10 of reprogramming (Figure S1D). These results confirmed that post-MET populations are also positively affected by TGF-β inhibition, underlining the multimodal nature of TGF-β signaling.

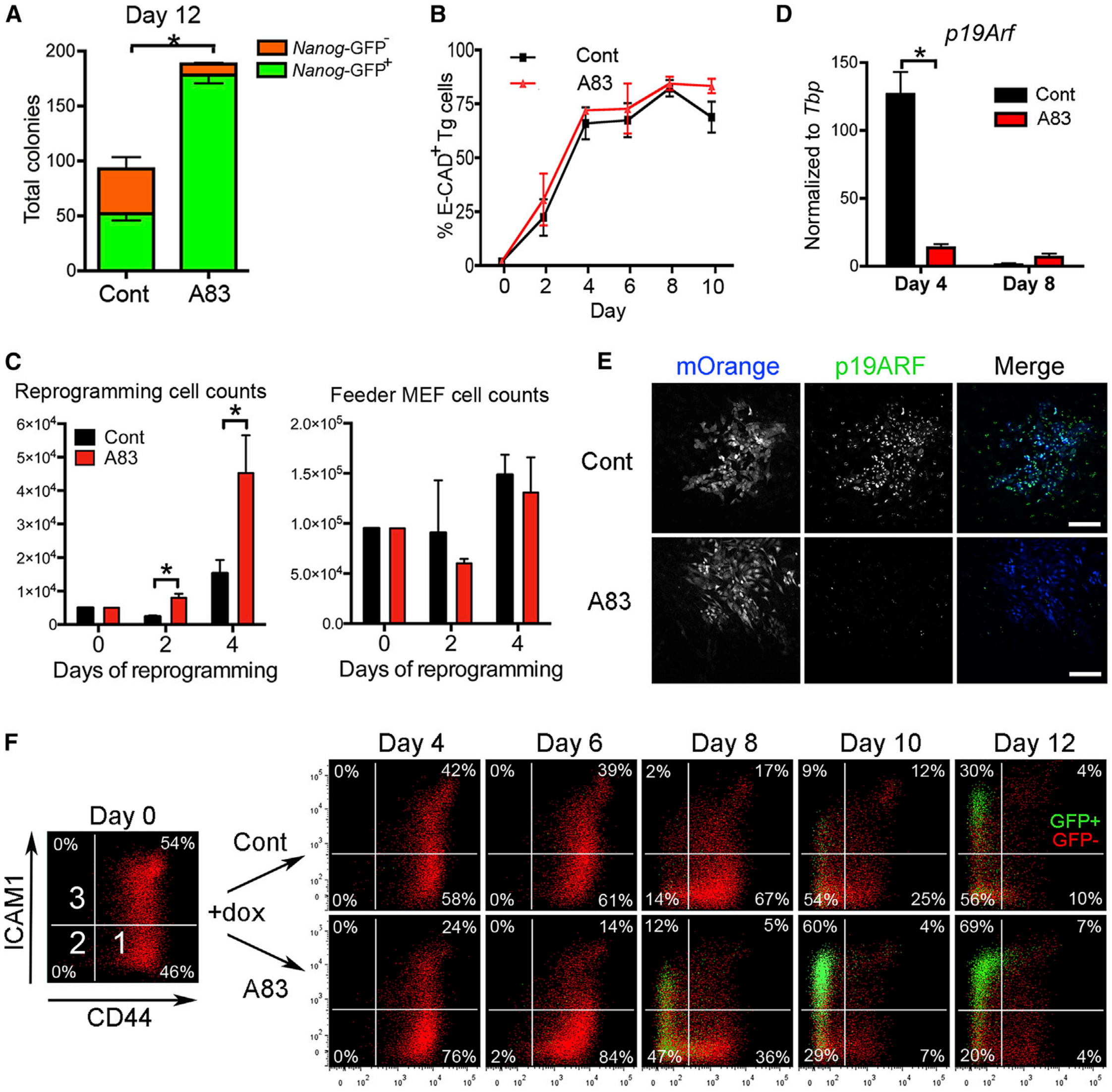

Figure 1. Reprogramming Enhancement and Acceleration by TGF-β Inhibitors

(A) *Nanog*-GFP⁻ and GFP⁺ colony numbers on day 12 of reprogramming.

(B) Flow cytometry analysis of E-CADHERIN (E-CAD) expression during reprogramming.

(C) Numbers of mOrange⁺ MKOS expressing transgenic (Tg) cells (left) and mOrange⁻ wild-type feeder MEFs (right) during the first 4 days.

(D) Real-time RT-PCR for *p19Arf* with mOrange⁺ cells on days 4 and 8.

(E) Immunofluorescence for p19ARF on day 4.

(F) CD44/ICAM1/*Nanog*-GFP expression throughout reprogramming. Red, *Nanog*-GFP⁻; green, *Nanog*-GFP⁺ Tg cells.

Cont and A83 indicate in the absence and presence of A83, respectively. See also Figure S1.

Increased Phosphorylated Smad3 Levels Correlate with Successful Reprogramming

Downstream targets of TGF-βRs include SMAD2/3, mitogen activated protein kinase (MAPK), Rho GTPase, and the phosphatidylinositol 3-kinase (PI3K)/AKT pathways (Derynck and Zhang, 2003; Zhang, 2009). TGF-βRs phosphorylate the C terminus of SMAD3, which causes complex formation with SMAD4 and translocation to the nucleus to regulate gene expression (Shi and Massagué, 2003). In the absence of A83 some, but not all, cells in reprogramming colonies demonstrated phosphorylated SMAD3 (p-SMAD3) at day 4 (Figure 2A, Cont Day4). p-SMAD3 was often observed in cells with *Nanog*-GFP expression at day 8 (Cont Day 8, Figure 2A), in contrast to day 12 when p-SMAD3 was barely detectable in *Nanog*-GFP⁺ cells (Cont Day 12, Figure 2A). Unexpectedly, we observed p-SMAD3⁺ cells more frequently within the colonies in the presence of A83 on both day 4 and day 8 of reprogramming (Figure 2A, A83). The finding that p-SMAD3⁺ cells increase in the presence of A83 contradicts the knowledge that ALK4/5/7 inhibitors dampen SMAD3 signaling (Tojo et al., 2005). However, the increase in p-SMAD3⁺ cells was also observed at day 4 of reprogramming with another ALK4/5/7 inhibitor, *SB*-431542 (SB43), which likewise enhances reprogramming (Ichida et al., 2009) (Figure 2B). When we quantified the percentage of mOrange⁺ Tg cells with p-SMAD3 foci in the whole-well images,

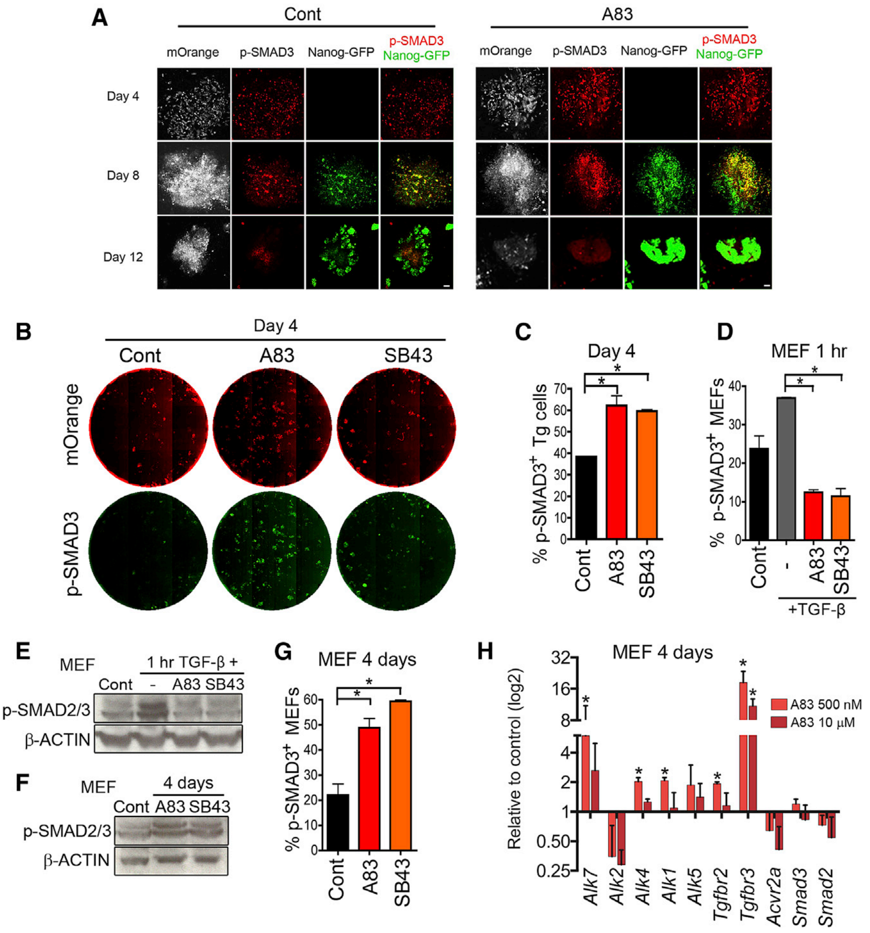

Figure 2. Increased Phosphorylated SMAD3 in the Presence of TGF-β Inhibitors

(A) Immunofluorescence for phosphorylated SMAD3 (p-SMAD3) on days 4, 8, and 12 of reprogramming in combination with the mOrange and *Nanog*-GFP reporters. Scale bar, 100 μm.

(B) Whole-well images of 6-well plates at day 4 of reprogramming. Red, mOrange$^+$ Tg cells; green, p-SMAD3 staining.

(C) Quantification of cells with strong p-SMAD3 foci in mOrange$^+$ Tg cells from the day 4 whole-well images.

(D) Whole-well p-SMAD3 image analysis with wild-type MEFs cultured for 1 hr with no treatment (Cont) or with the addition of 10 ng/mL TGF-β without inhibitors (–) or with A83-01 (A83) or SB431542 (SB43).

(E) p-SMAD2/3 western blotting in wild-type MEFs treated with 10 ng/mL TGF-β for 1 hr without inhibitors (–) or with A83 or SB43.

(F) p-SMAD2/3 western blotting in wild-type MEFs cultured for 4 days without inhibitors (Cont) or with A83 or SB43.

(G) Whole-well p-SMAD3 image analysis of wild-type MEFs cultured for 4 days without inhibitors (Cont) or with A83 or SB43.

(H) Real-time RT-PCR for *TGFβRs* and *Smad2/3* after 4 days culture of MEFs in the presence of A83. Each expression value was normalized to *Tbp* and then compared to DMSO-(carrier)-treated control samples.

All graphs represent averages of 3 independent experiments, with 2 technical replicates. Error bars indicate SD. *$p < 0.05$ based on a two-sided t test. See also Figure S2.

there was a 1.5-fold increase in the presence of either A83 or SB43 (Figures 2C and S2A). Notably, both the image analysis and western blotting demonstrated that either A83 or SB43 could block SMAD2/3 phosphorylation in MEFs treated with TGF-β for 1 hr (Figures 2D, 2E, and S2B). These data demonstrate that these inhibitors can work as predicted for short-duration treatments. However, when wild-type MEFs were cultured in the presence of inhibitors for 4 days, we observed a clear increase in p-SMAD2/3 levels (Figures 2F, 2G, and S2C) similar to that observed in Tg cells during reprogramming (Figures 2A–2C). Many TGF-βR (*Alk1*, *Alk4*, *Alk7*, *Tgfbr2*, and *Tgfbr3*) were upregulated in MEFs after 4 days-treatment with A83 (Figure 2H). This may be a feedback mechanism that contributes to the unexpected cellular response of increased p-SMAD2/3 levels during prolonged exposure to TGF-βR inhibitors.

Constitutively Active SMAD2/3 Boost Reprogramming

It was previously shown that SMAD3 is recruited to target loci by cell-type-specific master TFs, including by OCT4 to pluripotency gene loci in mouse ESCs (Mullen et al., 2011). Furthermore, SMAD3 interacts with several TFs, chromatin remodelers, and transcriptional regulators in a number of diverse cell types (Gaarenstroom and Hill, 2014). Our observations that the majority of cells becoming *Nanog*-GFP$^+$ are p-SMAD3$^+$ (Figure 2A) and prolonged treatment of cells with TGF-βR inhibitors increased the number of p-SMAD3$^+$ cells (Figures 2B–2G) prompted us to investigate roles for SMAD3 during reprogramming. We first tested whether constitutively active forms of SMAD2 (SMAD2CA) or SMAD3 (SMAD3CA) (Chipuk et al., 2002; Funaba and Mathews, 2000) could boost reprogramming. Interestingly, retroviral overexpression of *Smad2CA* and/or *Smad3CA* in our MKOS reprogramming system resulted in an over 6-fold increase in *Nanog*-GFP$^+$ colonies, and the combined effect of *Smad2CA* and *3CA* resulted in a 10-fold increase in efficiency (Figures 3A and S3A). Flow cytometry analysis revealed that expression changes of CD44, ICAM1, and *Nanog*-GFP were accelerated in the presence of *Smad3CA*, while E-CAD expression was not affected (Figures 3B–3D). Analyses of other reprogramming cell surface markers including MEFSK4, CD47, CD73 and CD104 (Lujan et al., 2015) also demonstrated accelerated reprogramming phenotypes at later time points beyond day 6 (Figure S3B). *Smad3CA* did not enhance the proliferation of cells undergoing reprogramming at the early stages (Figure 3E), different from A83 treatment (Figure 1C). When directly compared, reprogramming efficiency with A83 was higher than that of *Smad3CA* overexpression, and treatment with A83 and *Smad3CA* together did not further improve reprogramming efficiency (Figures 3F and S3C). The strong effect of A83, including its anti-senescence action, is potentially masking the effect of *Smad3CA* and/or their downstream mechanisms of facilitating

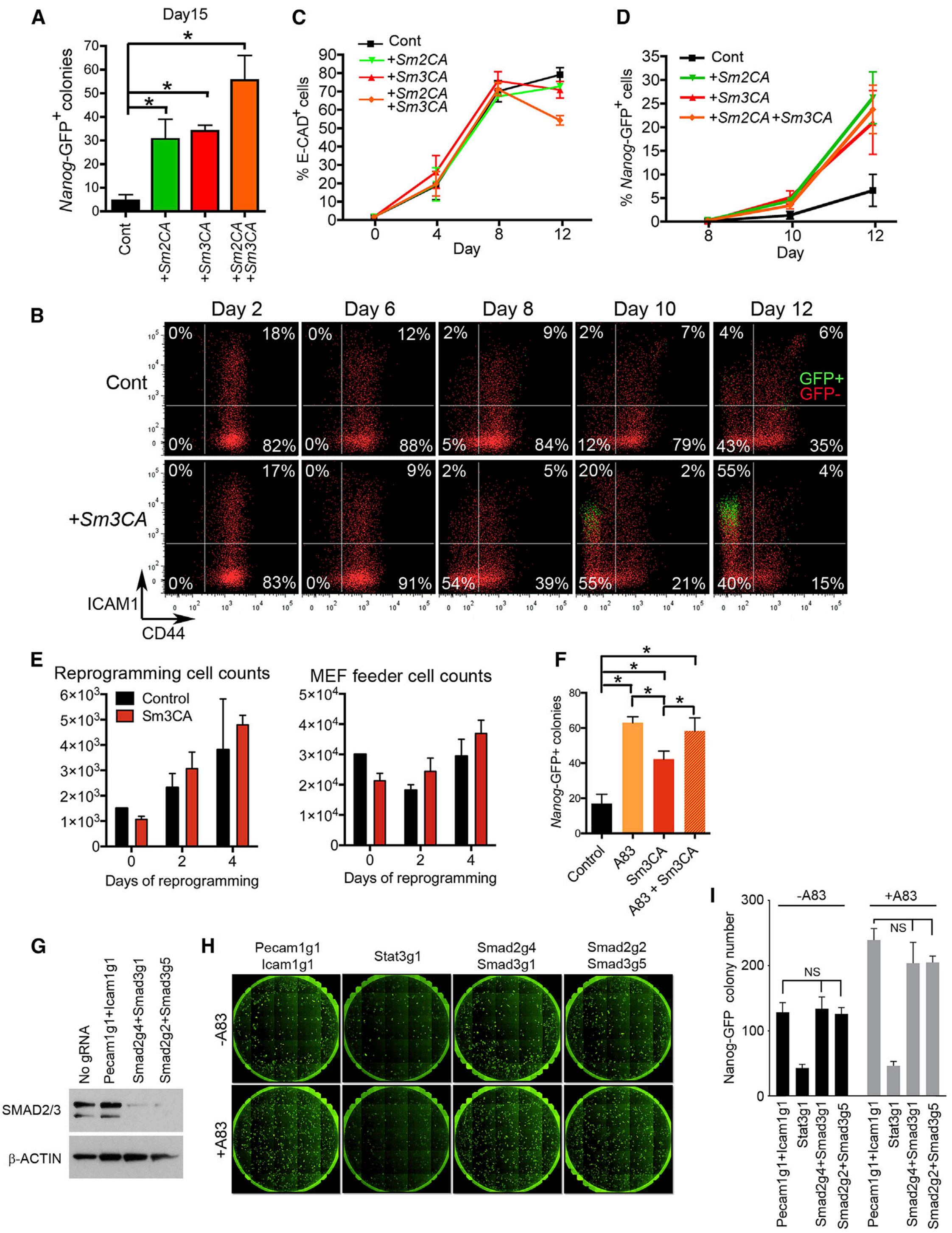

Figure 3. Constitutively Active Smad2/3 Boosts Reprogramming

(A) *Nanog*-GFP^{+} colony numbers on day 15 of reprogramming with retroviral infection of control vector (Cont), constitutive active *Smad2* (+*Sm2CA*), *Smad3* (+*Sm3CA*), or *Smad2CA* plus *Smad3CA* (+*Sm2CA*+*Sm3CA*).

(B) CD44/ICAM1/*Nanog*-GFP expression during reprogramming with control (top) and *Smad3CA* (bottom) expression vector infection. Red, *Nanog*-GFP^{-}; green, *Nanog*-GFP^{+} Tg cells.

(C and D) E-CAD (C) and *Nanog*-GFP (D) expression during reprogramming with control, *Smad2CA*, *Smad3CA*, or *Smad2CA* plus *Smad3CA* expression vector infection.

(E) Numbers of Tg (left) and wild-type feeder MEFs (right) with control (Cont) or *Sm3CA* vectors during reprogramming.

(F) *Nanog*-GFP^{+} colony numbers on day 15 of reprogramming in the presence of A83, *Sm3CA*, or A83 plus *Sm3CA*.

(legend continued on next page)

reprogramming overlap. To address whether A83-mediated reprogramming enhancement is attributed to the unexpected increase of p-SMAD2/3, we performed reprogramming after knocking out both *Smad2* and *Smad3* in dox-inducible MKOS MEFs with constitutive Cas9 expression by infection of lentiviral guide RNA (gRNA) expression vectors (Figure S3D) (Tzelepis et al., 2016). Efficient double knockout (KO) was confirmed by western blotting 3 days after gRNA vector infection (Figure 3G). Unexpectedly, *Smad2/3* double KO did not have obvious effects on reprogramming efficiency in either the presence or absence of A83 (Figures 3H and 3I). This indicated that reprogramming enhancement by A83 was largely SMAD2/3 independent and that endogenous SMAD2/3 is not required for mouse iPSC generation. Nevertheless, SMAD2/3CA also enhanced the generation of human iPSCs within an episomal reprogramming system (Okita et al., 2011) (Figure S3E). Independently, Yamakawa et al. (2016) also identified SMAD2 as one factor that can enhance human iPSC generation in a cDNA overexpression screen. These results solidified the notion that exogenous SMAD2/3 facilitates reprogramming and warranted further mechanistic analysis.

Accelerated Reprogramming Process by Smad3CA

First, we performed time-course global gene expression profiling. Day 15 *Nanog*-GFP$^+$ cells generated from reprogramming with the addition of *Smad3CA* were similar to ESCs (R^2 = 0.968), indicating that *Smad3CA* expression does not alter the final outcome of reprogramming (Figure S4A). When we compared samples on days 3, 6, 8, and 10 of reprogramming with and without *Smad3CA* expression, higher *Smad3* expression was clearly detectable in +*Smad3CA* samples, especially at earlier time points before viral vector silencing occurs (Figure 4A, red triangles). Unexpectedly, global gene expression was very similar between control and +*Smad3CA* samples across these time points (Figure 4A, gray dots). However, expression of almost all pluripotency-associated genes became higher in +*Smad3CA* samples by day 10, as further illustrated by gene set enrichment analysis (GSEA) (Figures 4A, 4B, and S4B; all read counts are available in Table S1) (Subramanian et al., 2005). Similarly, when we investigated expression patterns of genes >4-fold up- or downregulated in ESCs compared to MEFs (438 and 815 genes, respectively) during reprogramming, a small but global acceleration of up- and downregulation by Smad3CA was apparent (Figures 4C–4E). These results indicated that SMAD3CA expression did not cause outstanding gene expression changes compared to those induced by Yamanaka factors alone but globally boosted the required gene expression changes, resulting in a clear enhancement of reprogramming.

To further investigate how SMAD3CA altered the gene expression changes caused by Yamanaka factors, we first performed pattern clustering with control and +*Smad3CA* samples (Figure S4C). We classified 3,750 genes with above-background expression levels (mean count ≥ 2) and high variation (coefficient of variation >100) into 8 distinct gene expression patterns (scaled expression profiles are shown in Figure S4C, and classification of genes is available in Table S1), of which 7 cluster patterns in control and +*Smad3CA* sample series were very similar: downregulated by day 3 (Dwn), transiently upregulated at day 3 (D3Up), transiently upregulated at day 8 (D8Up), highly (D10Up) or slightly (D10sUp) upregulated at day 10 but low expression in ESCs, expected to be upregulated after day 10 (Up), and finally a down and up cluster (DwnUp). Additionally, the control series had a cluster of transiently upregulated genes on day 6 (D6Up), and the +*Smad3CA* series had a group of genes upregulated earlier than the Up genes (eUp). A comparison of the genes classified in each category revealed that genes in Dwn, D3Up, Up, and DwnUp clusters were very similar between control and +*Smad3CA* samples as shown with pale colors in the cord diagram (Figure S4D). Most of the genes in other categories were cross-classified between the control and +*Smad3CA* series. Among the cross-classified gene groups, 5 had over 100 genes (Figure S4D, *E-I). Most of the control D8Up genes were classified into +*Smad3CA* D10sUp genes (108 genes), with their upregulation minimized in the presence of +*Smad3CA* when medians of all the 108 genes from the control (blue) and +*Smad3CA* (red) series were plotted along the time points (Figure S4E). These 108 genes were enriched in nervous-system-related genes, potentially indicating unnecessary upregulation of SOX2 targets reflecting its critical roles in neurons (Figure S4J). Approximately 24% and 45% of control D10Up genes were cross classified into D8Up (124 genes) or D10sUp (233 genes) +*Smad3CA* clusters, where the peak of the transient upregulations of the median line in +*Smad3CA* occurred earlier at day 8 (Figure S4F) or was minimized (Figure S4G), respectively. These two groups of genes were enriched in immune-response-associated genes (Figure S4J). Most of the control D10sUp genes fell into +*Smad3CA* D10Up cluster (102 genes), but those had relatively low upregulation in the +*Smad3CA* series (compare +*Sm3CA* [red] median line in Figure S4H with the Cont [blue] median lines in Figures S4F and S4G). Finally, almost all genes that belonged to eUp, a unique cluster in the +*Smad3CA* series, were classified in the control Up cluster (120 genes) (Figure S4I), indicating an accelerated upregulation for these genes. Altogether, these gene expression cluster analyses suggested a global and swifter transition to a pluripotent state with less transient upregulation of miscellaneous genes and/or less production of divergent cells that are unable to become iPSCs in the presence of *Smad3CA*.

SMAD3 Interacts with Both Nucleosome Remodelers and Reprogramming Factors and Co-occupies OCT4 Targets throughout the Genome

SMAD3 is ubiquitously expressed and interacts with many different proteins, including TFs and co-activators, in various

(G) Efficient double knockout (dKO) of *Smad2* and *Smad3* was observed 72 hr after co-infection of lentiviruses encoding gRNA targeting *Smad2* and *Smad3* (Smad2g2+Smad3g1, Smad2g4+Smad3g5). Controls included samples with no gRNA infection and with co-infection of gRNA viruses against *Pecam1* (Pecam1g1) and *Icam1* (Icam1g1), respectively.

(H and I) *Smad2/3* double KO did not affect reprogramming efficiency in the presence or absence of A83. Pecam1g1+Icam1g1 and Stat3g1 (gRNA against *Stat3*) were used as negative and positive controls, respectively.

Graphs represent averages of 3 (A, C, D–F, F) or 2 (I) independent experiments with 2 technical replicates. Error bars indicate SD. *p < 0.05; NS, not significant (two-sided t test). See also Figure S3.

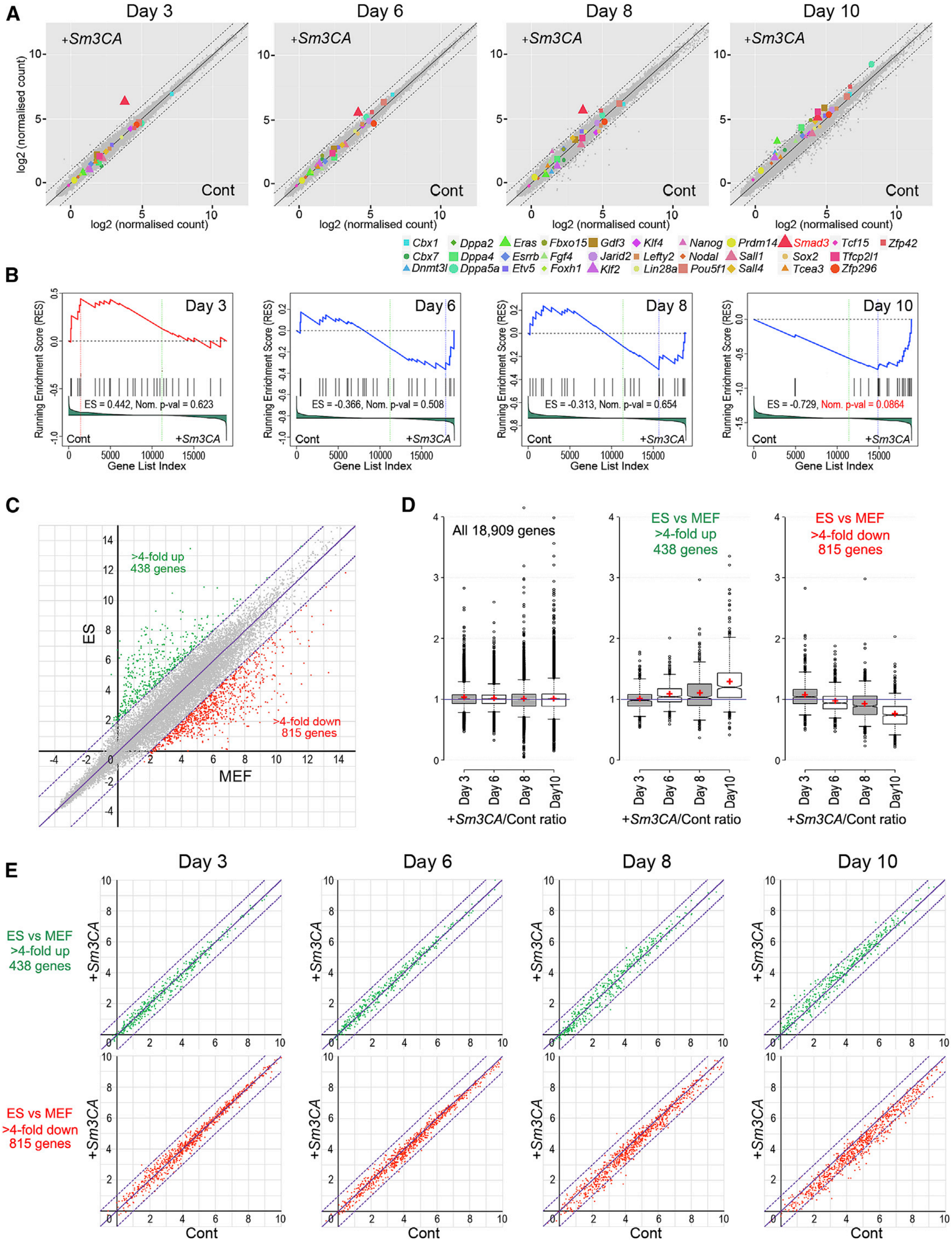

Figure 4. Accelerated Global Gene Expression Changes toward iPSCs by *Smad3CA*

(A) Global gene expression comparison between reprogramming with control (Cont) and +*Smad3CA* (+*Sm3CA*) vector expression on days 3, 6, 8, and 10. The diagonal dashed lines represent 2-fold differences.

(B) Gene set enrichment analysis (GSEA) for the pluripotency genes highlighted in (A). ES, enrichment score.

(C) Genes >4-fold up- and downregulated in ESCs compared to MEFs. Solid and dotted diagonal lines represent 1-fold and 4-fold changes, respectively. The x and y axes show log2 read counts.

(legend continued on next page)

cell types (Massagué et al., 2005). Its DNA-binding affinity is weak compared to other TFs, to the extent that its interaction with other TFs determines where SMAD3 binds throughout the genome (Massagué et al., 2005; Mullen et al., 2011; Shi et al., 1998). We therefore hypothesized that SMAD3, in combination with nucleosome remodelers or co-activators, is recruited by OCT4, SOX2, and/or KLF4 to their binding sites, which augments expression of target genes during reprogramming. Indeed, SMAD3 immunoprecipitation pulled down OCT4, SOX2, and KLF4 (mildly) at day 4 of reprogramming when expressing *Smad3CA* (Figure 5A). We could also detect interactions between SMAD3 and histone modifiers and transcriptional regulators, including DPY30 (part of the MLL complex), p300, and BRG1 (part of the SWI/SNF complex), but not MED15 (part of the Mediator complex) (Figure 5A). To investigate the possibility that SMAD3 bridges reprogramming factors with co-activators, we assessed interactions between OCT4 and the co-activators at day 4 of reprogramming in the presence or absence of *Smad3CA*. Interaction between OCT4 and DPY30 (MLL complex), but not p300, was readily detectable at day 4 of reprogramming in the control conditions (Figure 5B). However, when SMAD3CA was overexpressed, the amount of DPY30 pulled down with OCT4 increased nearly 3-fold (Figures 5B and 5C), indicating that SMAD3CA facilitated the interaction between OCT4 and the MLL complex component. Furthermore, ChIP-seq analysis revealed that just 3 days of MKOS induction largely altered SMAD3-binding sites across the genome, yielding 1,176 novel binding sites and losing the 904 binding sites observed in the starting MEF population (false discovery rate [FDR] < 0.1) (Figure 5D). The Oct4-binding sites on day 3 of reprogramming were clearly co-occupied by SMAD3, while SMAD3 binding was hardly detectable in the absence of Yamanaka factor expression at those loci, suggesting a recruitment of SMAD3 by MKOS (Figures 5E and 5F). Chromatin at those OCT4-binding loci was closed in MEFs, but open on day 3 of reprogramming either in the presence or absence of exogenous *Smad3CA* (Figures 5E and 5F), confirming OCT4 as a pioneering factor (Soufi et al., 2012). We then performed an assay for transposase accessible chromatin with high-throughput sequencing (ATAC-seq), which demonstrated that chromatin at early OCT4-binding loci was similarly accessible regardless of either the presence or absence of SMAD3CA co-expression on day 3 (Figures 5E and 5F). It has been reported that histone marks of OCT4 target loci dynamically change during reprogramming (Chronis et al., 2017). Considering the ability of SMAD3 to interact with histone modifiers, such as Dpy30 and p300 (Figure 5A), SMAD3CA might contribute to such changes and facilitate gene expression changes toward iPSCs. In fact, we could detect increased H3K4me3 at promoters of 5 out of 6 pluripotency genes (Figure 5H, red), near which SMAD3 binding was observed on day 8 of reprogramming (blue, Figure 5G), although it could be either a cause or a consequence of enhanced reprogramming by SMAD3CA. In summary, these results support a model whereby Smad3 interacts with reprogramming factors and is directed to their target loci, which potentially facilitates the recruitment of histone modifiers and/or nucleosome remodelers, facilitating the cell identity change.

Smad3CA Potentiates Other Master-TF-Mediated Cell Identity Conversions

Before and after the initial generation of iPSCs in 2006, several cell types have been generated by overexpression of cell-type-specific master TFs (Bussmann et al., 2009; Davis et al., 1987; Feng et al., 2008; Kajimura et al., 2009; Pfisterer et al., 2011; Vierbuchen et al., 2010; Xie et al., 2004). SMAD3 is known to interact with a multitude of cell-type-specific master TFs (Massagué, 2012; Mullen et al., 2011). We asked whether *Smad2/3CA* could boost other master-TF-mediated cell identity conversions. B cells can be converted to macrophages by overexpression of *Cebpα* (Bussmann et al., 2009; Fukuchi et al., 2006; Xie et al., 2004) (Figure 6A). The conversion is near 100% efficient, occurring over a 6-day period, and can be monitored by downregulation of the B cell surface marker CD19 and upregulation of the macrophage marker MAC-1. Expression of *Smad2/3CA* hardly affected CD19 downregulation; however, more than 75% of cells became MAC-1$^+$ by day 4 of conversion with *Smad2/3CA* compared to less than 30% of cells converted with *Cebpα* alone (Figures 6B and 6C). Furthermore, in the *Smad2/3CA* expressing samples, cells with very high granularity, a feature of mature myeloid cells, could be observed from day 3 (Figure 6D). Quantitative PCR (qPCR) detected accelerated upregulation of the macrophage markers *Mac1*, *Csfr*, and *Fcgr1* and downregulation of the pre-B cell markers *Rag1*, *Pax5*, and *Vpreb2* (Figure 6E). By analyzing CEBPα ChIP-seq data 18 hr after initiation of the B cell to macrophage conversion, we also found that 1,255 out of 4,211 CEBPα-binding peaks had SMAD-binding motifs (Figures S5A and S5B) (Di Stefano et al., 2016). Genes associated with those peaks were highly enriched in gene ontology terms related to macrophage functions (Figure S5C). ChIP-qPCR confirmed that SMAD3 indeed bound to these loci during the B cell to macrophage conversion (Figures S5D and S5E). In summary, SMAD2/3CA significantly accelerated and enhanced the CEBPα-mediated B cell to macrophage conversion, likely via its recruitment to CEBPα targets.

Next, we investigated the process of converting myoblasts to adipocytes with *Cebpβ* and *Prdm16* (Kajimura et al., 2009) (Figure 6F). Successful conversion of C2C12 myoblasts to adipocytes can be assessed by oil red O staining, which identifies the triglycerides and lipids in converted adipocytes. Expression of either *Smad2CA* or *Smad3CA* in conjunction with *Cebpβ* and *Prdm16* resulted in an ~20% increase in conversion efficiency (Figures 6G and 6H).

Finally, we addressed whether *Smad2/3CA* can accelerate conversion of human fibroblasts to neurons by *Ascl1*, *Brn2a*, *Mytll*, and *NeuroD1* (ABMN) (Figure 7A) (Pang et al., 2011). The proportion of cells with neuronal morphology and expressing the

(D) Read count ratios (+Sm3CA/Cont) of all 18,909 genes, 438 ESC upregulated genes, and 815 ESC downregulated genes at days 3, 6, 8, and 10 are shown with boxplots. Red crosses represent mean.

(E) Scatterplots (+Sm3CA versus Cont) of ESC up- and downregulated genes in (C) and (D). Solid and dotted diagonal lines represent 1-fold and 2-fold changes, respectively.

See also Figure S4 and Table S1.

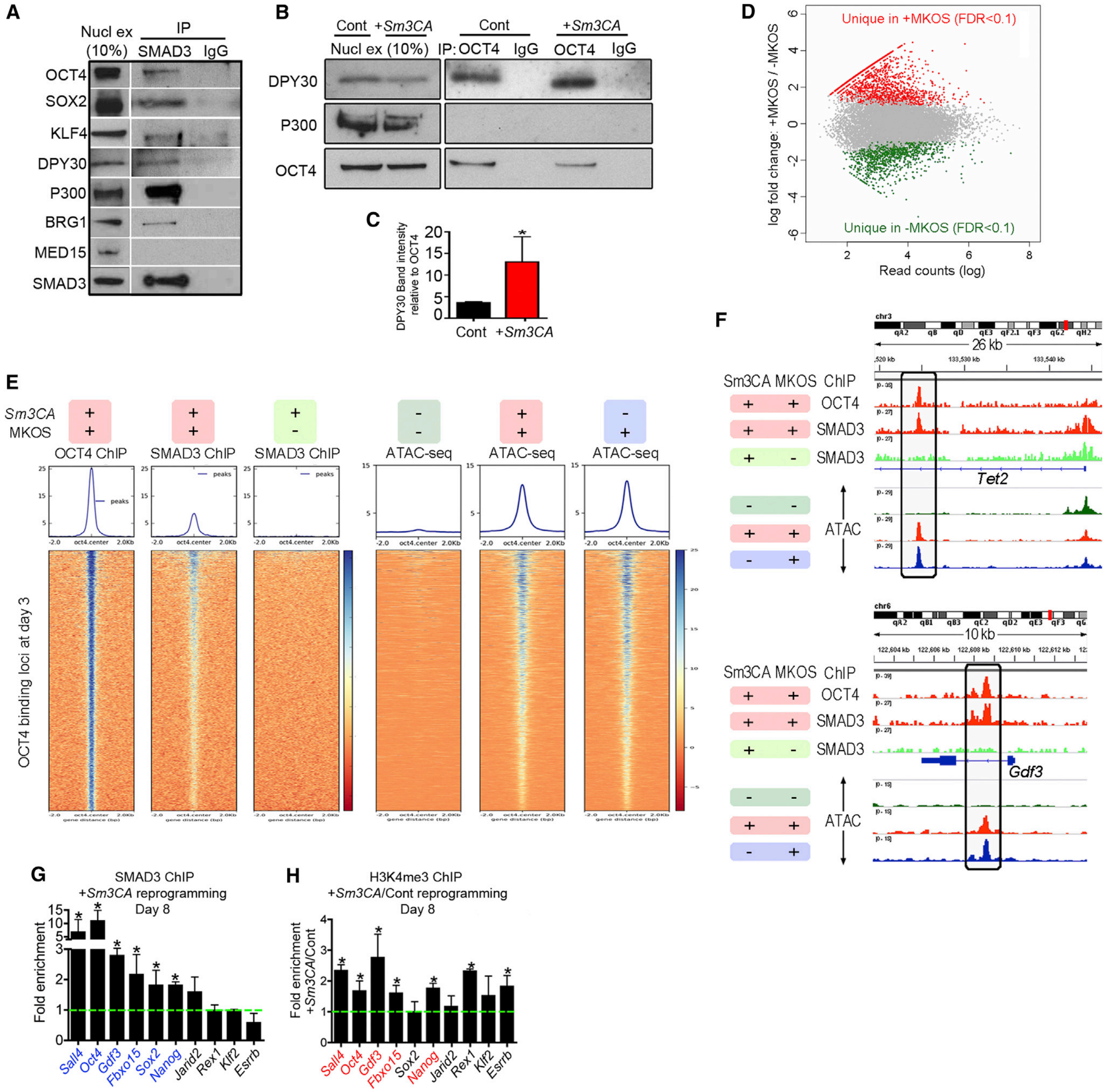

Figure 5. SMAD3 Interacts with Reprogramming Factors and Chromatin Remodelers and Is Localized at OCT4-Binding Sites during Reprogramming

(A) Co-immunoprecipitation (co-IP) of SMAD3 and reprogramming factors or chromatin remodelers at day 4 of reprogramming with *Smad3CA* expression. Nucl ex, 10% of nuclear extract used for co-IP; IgG, immunoglobulin G (negative control for co-IP).

(B) Co-IP of OCT4 and DPY30 at day 4 of reprogramming with control (Cont) or *Smad3CA* (+*Sm3CA*) vector expression. An interaction between OCT4 and P300 was not observed.

(C) DPY30 band intensity analysis from 2 independent experiments shown in (B).

(D) Comparison of SMAD3-binding peaks in MEFs with overexpression of *Sm3CA* or *Sm3CA* plus Yamanaka factors for 3 days.

(E and F) OCT4, SMAD3 ChIP-seq, and ATAC-seq heatmaps at OCT4 bound loci (E) and tracks at the *Tet2* and *Gdf3* loci (F) with (+) and without (–) MKOS induction in the presence (+) or absence of (–) Sm3CA expression for 3 days.

(G) SMAD3 ChIP-qPCR at day 8 of reprogramming with *Smad3CA* expression at known Oct4-binding loci in ESCs associated with pluripotency genes. Fold enrichment refers to enrichment over the averaged value of binding to known unbound regions of the *Oct4* and *Nanog* genes.

(H) H3K4me3 ChIP-qPCR at day 8 of reprogramming with control (Cont) and *Smad3CA* (+*Sm3CA*) vector expression. Data are shown as relative enrichment in +*Sm3CA* samples against control samples.

Graphs represent averages of 2 (H3K4 ChIP) or 3 (Smad3 ChIP) independent experiments with 2 technical replicates. Error bars indicate SD. *p < 0.05 (one-sided t test).

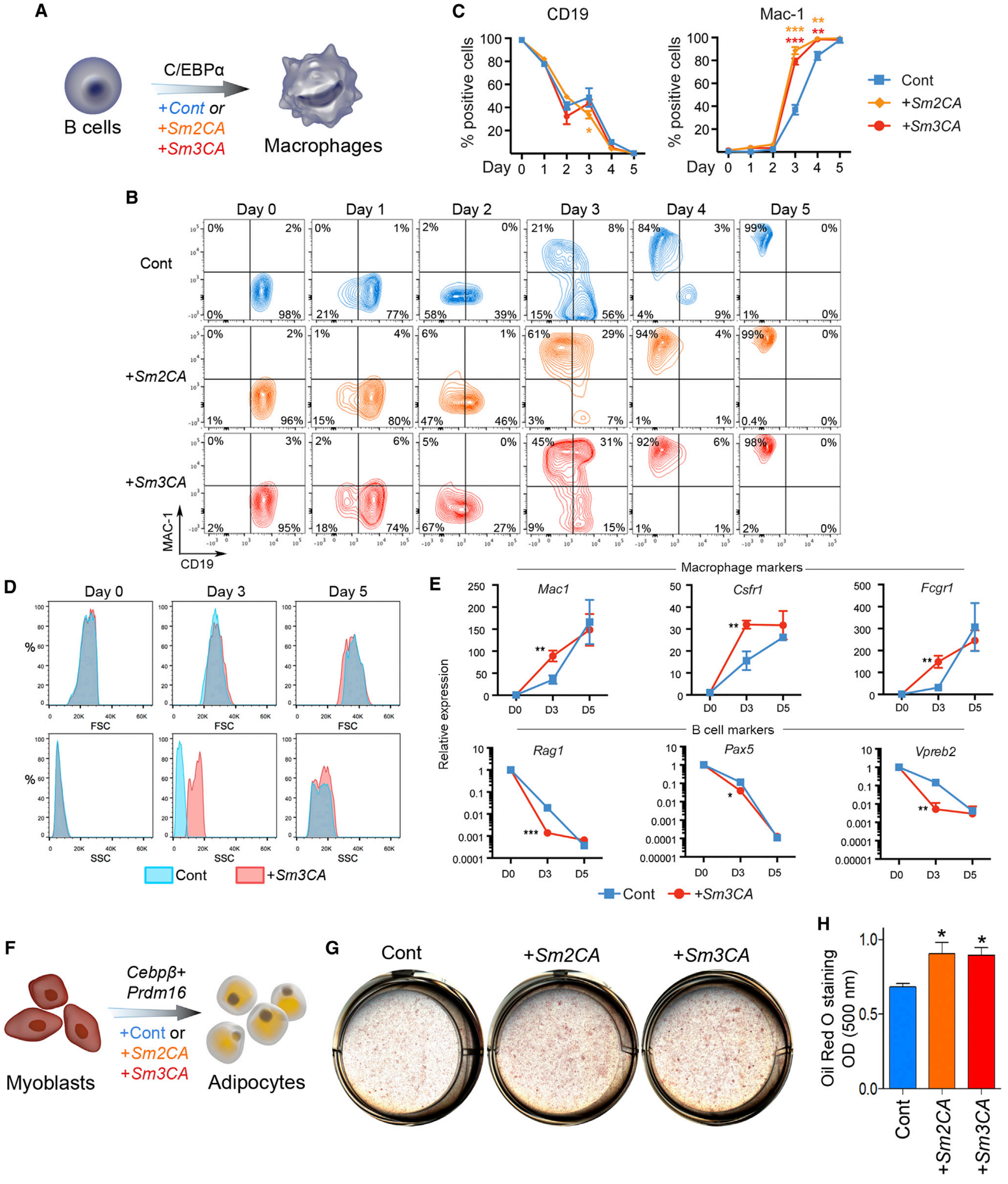

Figure 6. ***Smad2/3CA*** **Potentiate Macrophage and Adipocyte Conversions**

(A) Conversion of B cells to macrophages by retroviral transduction of *Cebpα* with or without *Smad2CA* (*Sm2CA*) or *Smad3CA* (*Sm3CA*).

(B) Expression changes of the B cell marker CD19 and the macrophage marker MAC-1 during the *Cebpα*-mediated transdifferentiation process with additional control (Cont), *Sm2CA*, or *Sm3CA* vector expression.

(C) Quantification of CD19 and MAC-1 expression.

(D) Changes in forward scatter (FSC) and side scatter (SSC).

(legend continued on next page)

neuronal lineage marker MAP2 was not affected by the overexpression of *Smad2*/3CA, as assessed at day 7, 12, or 20 after initiation of ABMN transgene expression (Figures 7B and 7C). However, we could observe a clear interaction between SMAD3 and one of the induced neuron (iN) factors, BRN2 (Figure 7D), and when we assessed neuronal maturity by patch-clamp electrophysiology, marked differences emerged (Figure 7E). On days 23–25 of the neural conversion, 25% (3/12) of human iNs (hiNs) generated with ABMN showed only immature single action potentials (APs) after step-wise current injections, and 75% (9/12) were not functional at this time point (Figure 7E, Cont). In contrast, with the addition of *Smad2CA* or *Smad3CA*, 44% (4/9) or 40% (6/15) of hiNs had single APs, and 33% (3/9) or 26.7% (4/15) showed a more mature phenotype with repetitive APs, respectively, which was consistent with their significantly greater membrane capacitance and Na^+, K^+ currents (Figures 7E, S6A, and S6B). To reveal molecular signatures that explain this accelerated maturation, we performed RNA sequencing (RNA-seq) analysis. To our surprise, gene expression was very similar on day 23, with only 5 differentially expressed genes (*USH1C*, *C12orf29*, *KIAA0391*, *SEZ6*, and *COMMD8* with fold change [Fc] >2, FDR <0.1) between control and +*Smad3CA* iNs (Figure 7F; all reads counts are available in Table S2). There was also no difference in expression of glutamatergic- and GABAergic-neuron-associated genes, which are the main neural subtypes generated by ABMN (Figure S6C) (Pang et al., 2011; Vierbuchen et al., 2010). However, earlier phenotypes were evident in 294 and 309 genes up- and downregulated, respectively, on day 12 in iNs generated with *Smad3CA* (Figure 7G; Fc >2, FDR <0.1). The most enriched gene ontology (GO) terms in the upregulated genes were "nucleobase-containing compound transport," "ectoderm development," and "nervous system development" genes (Figure 7H). Intriguingly, many of those genes are further upregulated on day 23 in both control and +*Smad3CA* (Figure 7H, pale blue and pink). Thus, we hypothesized that SMAD3CA expression accelerated gene expression changes caused by ABMN in a global manner, similar to what was observed in iPSC generation, and resulted in faster neural maturation. To test this hypothesis, we generated a list of differentially expressed genes between day 12 and day 23 control samples and identified 634 up- and 347 downregulated genes at day 23 (>3-fold, FDR <0.1) (Figure 7I). The most enriched GO terms in these upregulated genes were "cell communication," "biological adhesion," "ectoderm development," and "nervous system development" (Figure 7J). Strikingly, the majority of the 634 late iN upregulated genes were more highly expressed in +*Smad3CA* iNs on day 12 (Figure 7K). Global, faster downregulation of the late iN downregulated genes was also evident in day 12 +*Smad3CA* iNs, including multiple "metabolic process"-related genes (Figure 7L). These results revealed that when co-expressed with neural master TFs, SMAD3CA could remarkably accelerate global gene expression changes, resulting in faster iN maturation and formation of neural connectivity as revealed by patch-clamp recordings.

In summary, in addition to iPSC generation with Yamanaka factors, SMAD2/3CA potentiated three other master-TF-mediated cell identity conversions, showing that SMAD2/3 are partners of multiple TFs and chromatin modifiers in various contexts.

DISCUSSION

Cell identity conversions by the exogenous expression of master TFs offer powerful tools for tissue engineering, disease modeling, and regenerative medicine, yet a deeper understanding of the underlying molecular mechanisms is necessary to make such extraordinary processes more efficient and prompt. This study identified that exogenous, constitutive active SMAD2/3 can act as a part of the core cellular machinery that potentiates forced cell identity changes with master TFs. Mechanistically, during iPSC generation, SMAD3 co-occupied OCT4-binding sites across the genome and facilitated cellular conversion, likely providing a platform to recruit chromatin remodelers and transcriptional activators.

Our study commenced with the investigation of how TGF-βR inhibitors enhance reprogramming efficiency. One of the mechanisms involved suppression of p19ARF upregulation. While we observed that prolonged TGF-βR inhibitor treatment unexpectedly increased p-SMAD2/3 levels, double KO of endogenous *Smad2/3* did not affect reprogramming efficiency in the presence or absence of A83, indicating that the positive effects of A83 are likely independent of SMAD2/3. However, overexpression of constitutively active SMAD2/3 boosted iPSC generation through recruitment to Oct4 target loci and markedly enhanced three other master-TF-mediated cell conversions.

In addition to widely expressed co-activators and co-repressors, a multitude of cell-type-specific TFs interact with SMAD2/3 (Massagué et al., 2005), partially explaining cell-type-dependent responses to TGF-β signaling (Mullen et al., 2011). During cell fate specification, it is thought that TFs might compete to recruit active SMAD2/3-co-activator complexes to target loci and activate their targets (Massagué, 2012; Mullen et al., 2011). Analogously, the amount of active SMAD2/3 could be a rate-limiting factor to fully realize the exogenous TF's potential during forced cell identity changes, resulting in inefficient and incomplete cellular conversion, even when the best possible combinations of master TFs are used. In such cases, *SMAD2/3CA* overexpression might overcome these limitations and enhance cell conversions.

The rapid and efficient generation of functionally mature adult cell types represents a major challenge in the direct cellular

(E) qPCR analysis of macrophage and pre-B cell markers.

Graphs in (C) and (E) represent averages of 2 independent experiments with 2 technical replicates, respectively. *p < 0.05; **p < 0.01; ***p < 0.001 (two-sided t test).

(F) Conversion of C2C12 myoblasts to adipocytes with expression of *Cebpβ* and *Prdm16* with or without *Sm2CA* or *Sm3CA*.

(G) Oil red O staining of cells 6 days post induction of *Cebpβ* and *Prdm16* with an additional control (Cont), *Sm2CA*, or *Sm3CA* vector.

(H) Quantification of oil red O staining. Graphs represent averages of 2 independent experiments, with 3 technical replicates, one of those images is presented in G. **p < 0.01; *p < 0.05 (two-sided t test).

See also Figure S5.

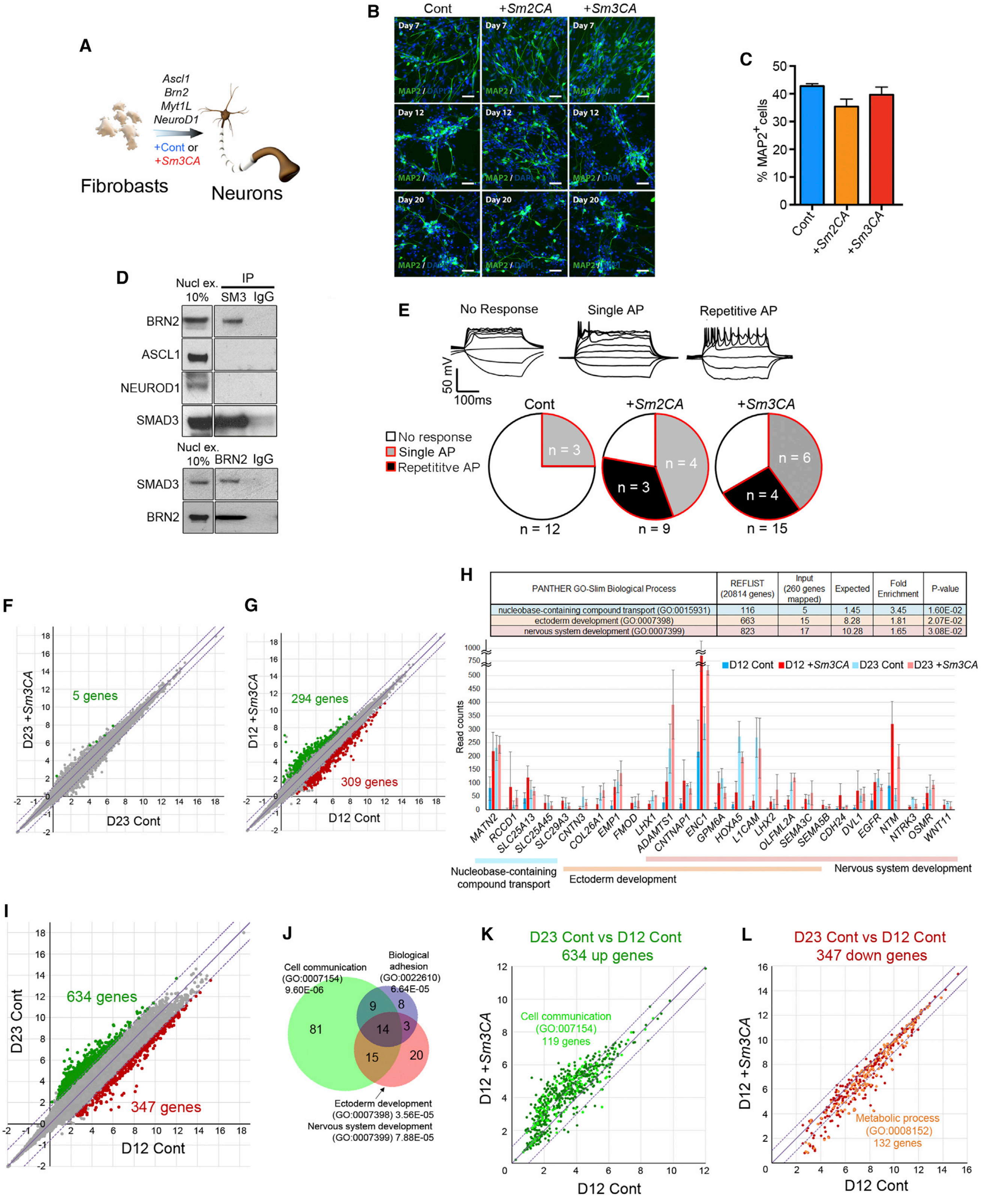

Figure 7. *Smad3CA* Accelerates the Generation of Mature iNs

(A) Conversion of human fibroblasts to iNs by lentiviral transduction of *Ascl1*, *Brn2a*, *Myt1L*, and *NeuroD1* (ABMN) with or without *Smad2CA* (*Smad2CA*) or *Smad3CA* (*Sm3CA*).

(legend continued on next page)

conversion and ESC differentiation fields. For example, most of the protocols used to generate fully functional human neurons, whether derived from ESCs or by direct conversion of differentiated cells, require months (Brennand et al., 2011; Kriks et al., 2011; Pereira et al., 2014). The use of SMAD2/3CA in the generation of mature neurons and potentially other challenging cell types represents an exciting opportunity for disease modeling and regenerative medicine. Various cell types share multiple TFs, co-activators, and co-repressors, and they contribute to the establishment of unique cellular character during normal development. Our findings propose that other shared co-factors could also be powerful tools to achieve more efficient and precise cell conversions.

STAR★METHODS

Detailed methods are provided in the online version of this paper and include the following:

- KEY RESOURCES TABLE
- CONTACT FOR REAGENT AND RESOURCE SHARING
- EXPERIMENTAL MODEL AND SUBJECT DETAILS
  - Primary cell lines
- METHOD DETAILS
  - MEF Reprogramming
  - B cell to macrophage transdifferentiation
  - Myoblast to adipocyte transdifferentiation
  - Generation of human iNs
  - Western Blotting
  - Plasmids
  - Fluorescence activated cell sorting
  - Immunofluorescence microscopy
  - Smad2/3 double KO MEF reprogramming
  - Retroviral mediated gene transduction for iPSC reprogramming enhancement
  - Human fibroblast reprogramming to iPSCs
  - RNA-sequencing and data analysis
  - Co-immunoprecipitation
  - Chromatin Immunoprecipitation followed by high-throughput DNA sequencing (ChIP-seq)
  - Nuclear isolation and sonication
  - Immunoprecipitation and DNA isolation
  - ChIP library preparation for sequencing
  - Adding A bases to 3′ end of DNA fragment
  - ChIP-seq data analyses
  - ATAC-seq
  - ATAC-seq data analysis
  - ChIP-qPCR for reprogramming to iPSCs
  - qPCR in B cell to macrophage transdifferentiation
  - Bioinformatics analysis of B cell to macrophage transdifferentiation ChIP-seq data
- QUANTIFICATION AND STATISTICAL ANALYSES
- DATA AND SOFTWARE AVAILABILITY

SUPPLEMENTAL INFORMATION

Supplemental Information includes six figures and three tables and can be found with this article online at https://doi.org/10.1016/j.stem.2017.10.013.

AUTHOR CONTRIBUTIONS

T.R. performed all iPSC reprogramming experiments. B.D.S., T.V.T., and S.C. contributed to the macrophage and adipocyte conversion experiments. U.P. and D.R.O. conducted iN experiments. J.A. and J.R.M. analyzed ChIP-seq, ATAC-seq, and RNA-seq data. E. Chantzoura generated the TNG MKOS ESC line. E. Cohen performed cloning for human cell reprogramming and optimized co-immunoprecipitation methods. M.B., D.F.K., L.T., W.T., and K.Y. contributed to the Cas9-mediated KO experiments. A.J. and S.L. performed multiplexed RNA-seq. T.R., T.G., M.P., and K.K. conceived the study and wrote the manuscript.

ACKNOWLEDGMENTS

We thank F. Rossi and C. Cryer for assistance with flow cytometry, M. Foronda for comments on the manuscript, and B. Mattsson for help with illustrations. We also thank L. Robertson and J. Rennie for technical assistance and the Biomed unit staff for mouse husbandry. We thank Dr. Hideaki Nakajima for the C/EBPaER mice. This work was supported by the ERC (grants ROADTOIPS 261075 to K.K. and BRAINCELL 261063 to S.L. and iN-Brain 309712 to M.P.), the BBSRC (project grant BB/L023474/1 to K.K.), the Anne Rowling Regenerative Neurology Clinic (K.K.), the Swedish Research Council (grant STARGET to S.L. and grants 521-2012-5624 and 521-2013-3347 to M.P.), and the Wellcome Trust (grant WT098051 to K.Y.). T.R., L.T., J.A., and D.F.K. are funded by a Darwin Trust scholarship, a CMVM scholarship, and Principal's Career Development scholarship from the University of Edinburgh, respectively. D.F.K. is supported by the BBSRC (EASTBIO doctoral training partnership). T.V.T. is supported by a Juan de la Cierva postdoctoral fellowship (MINECO, FJCI-2014-22946). B.D.S. was supported by an EMBO long-term fellowship (ALTF 1143-2015). K.K. is an MRC senior non-clinical fellow (MR/N008715/1). M.P. is a New York Stem Cell Foundation Robertson Investigator. We acknowledge support from the Spanish Ministry of Economy and Competitiveness, Centro de Excelencia Severo Ochoa 2013-2017, and the CERCA Program Generalitat de Catalunya.

Received: January 16, 2016
Revised: July 17, 2017
Accepted: October 25, 2017
Published: November 22, 2017

(B) Confocal imaging of the neural marker MAP2 7, 12, and 20 days after viral infection with *ABMN* (Cont) or *ABMN+Sm2CA or Sm3CA*. Scale bars, 50 μm.

(C) Quantification of MAP2+ cells (% of total cells) on day 12. Data represent averages of 2 independent experiments with 2 technical replicates.

(D) Co-immunoprecipitation of BRN2 and SMAD3 when overexpressed in HEK293 cells.

(E) Electrophysiological measurements using whole-cell patch clamp at day 23–25 post-conversion initiation using *ABMN* (Cont) or *ABMN+Sm2CA* or *Sm3CA*. Top panels represent traces of current-induced action potentials (APs) of different phenotypes.

(F and G) Gene expression comparison between flow-sorted NCAM1^{+} *ABMN* (Cont) and *ABMN+Sm3CA* iNs on day 23 (F) and day 12 (G). The x and y axis show log2 read counts; green and red genes, fold change (Fc) >2, FDR <0.1.

(H) Expression of genes that have the top 3 most enriched gene ontology (GO) terms in the 294 upregulated genes in day 12 +*Sm3CA* iNs.

(I) Differentially expressed genes between day 12 and day 23 iNs with *ABMN* (Cont) (Fc >3, FDR < 0.1).

(J) Top 4 most enriched GO terms in the 634 upregulated genes on day 23, and number of genes with these GO terms.

(K and L) Expression comparison of up-regulated (K) and down-regulated (L) genes highlighted in (I) between *ABMN* (Cont) and *ABMN+Sm3CA* iNs on day 12. See also Figure S6 and Table S2.

REFERENCES

Bertero, A., Madrigal, P., Galli, A., Hubner, N.C., Moreno, I., Burks, D., Brown, S., Pedersen, R.A., Gaffney, D., Mendjan, S., et al. (2015). Activin/nodal signaling and NANOG orchestrate human embryonic stem cell fate decisions by controlling the H3K4me3 chromatin mark. Genes Dev. *29*, 702–717.

Bolger, A.M., Lohse, M., and Usadel, B. (2014). Trimmomatic: a flexible trimmer for Illumina sequence data. Bioinformatics *30*, 2114–2120.

Brennand, K.J., Simone, A., Jou, J., Gelboin-Burkhart, C., Tran, N., Sangar, S., Li, Y., Mu, Y., Chen, G., Yu, D., et al. (2011). Modelling schizophrenia using human induced pluripotent stem cells. Nature *473*, 221–225.

Buenrostro, J.D., Giresi, P.G., Zaba, L.C., Chang, H.Y., and Greenleaf, W.J. (2013). Transposition of native chromatin for fast and sensitive epigenomic profiling of open chromatin, DNA-binding proteins and nucleosome position. Nat. Methods *10*, 1213–1218.

Bussmann, L.H., Schubert, A., Vu Manh, T.P., De Andres, L., Desbordes, S.C., Parra, M., Zimmermann, T., Rapino, F., Rodriguez-Ubreva, J., Ballestar, E., and Graf, T. (2009). A robust and highly efficient immune cell reprogramming system. Cell Stem Cell *5*, 554–566.

Chambers, I., Silva, J., Colby, D., Nichols, J., Nijmeijer, B., Robertson, M., Vrana, J., Jones, K., Grotewold, L., and Smith, A. (2007). Nanog safeguards pluripotency and mediates germline development. Nature *450*, 1230–1234.

Chantzoura, E., Skylaki, S., Menendez, S., Kim, S.I., Johnsson, A., Linnarsson, S., Woltjen, K., Chambers, I., and Kaji, K. (2015). Reprogramming roadblocks are system dependent. Stem Cell Reports *5*, 350–364.

Cheloufi, S., Elling, U., Hopfgartner, B., Jung, Y.L., Murn, J., Ninova, M., Hubmann, M., Badeaux, A.I., Euong Ang, C., Tenen, D., et al. (2015). The histone chaperone CAF-1 safeguards somatic cell identity. Nature *528*, 218–224.

Chen, X., Xu, H., Yuan, P., Fang, F., Huss, M., Vega, V.B., Wong, E., Orlov, Y.L., Zhang, W., Jiang, J., et al. (2008). Integration of external signaling pathways with the core transcriptional network in embryonic stem cells. Cell *133*, 1106–1117.

Chipuk, J.E., Cornelius, S.C., Pultz, N.J., Jorgensen, J.S., Bonham, M.J., Kim, S.J., and Danielpour, D. (2002). The androgen receptor represses transforming growth factor-beta signaling through interaction with Smad3. J. Biol. Chem. *277*, 1240–1248.

Chronis, C., Fiziev, P., Papp, B., Butz, S., Bonora, G., Sabri, S., Ernst, J., and Plath, K. (2017). Cooperative binding of transcription factors orchestrates reprogramming. Cell *168*, 442–459.

Davis, R.L., Weintraub, H., and Lassar, A.B. (1987). Expression of a single transfected cDNA converts fibroblasts to myoblasts. Cell *51*, 987–1000.

Derynck, R., and Zhang, Y.E. (2003). Smad-dependent and Smad-independent pathways in TGF-beta family signalling. Nature *425*, 577–584.

Di Stefano, B., Sardina, J.L., van Oevelen, C., Collombet, S., Kallin, E.M., Vicent, G.P., Lu, J., Thieffry, D., Beato, M., and Graf, T. (2014). C/EBPα poises B cells for rapid reprogramming into induced pluripotent stem cells. Nature *506*, 235–239.

Di Stefano, B., Collombet, S., Jakobsen, J.S., Wierer, M., Sardina, J.L., Lackner, A., Stadhouders, R., Segura-Morales, C., Francesconi, M., Limone, F., et al. (2016). C/EBPα creates elite cells for iPSC reprogramming by upregulating Klf4 and increasing the levels of Lsd1 and Brd4. Nat. Cell Biol. *18*, 371–381.

Feng, X.H., Zhang, Y., Wu, R.Y., and Derynck, R. (1998). The tumor suppressor Smad4/DPC4 and transcriptional adaptor CBP/p300 are coactivators for smad3 in TGF-beta-induced transcriptional activation. Genes Dev. *12*, 2153–2163.

Feng, R., Desbordes, S.C., Xie, H., Tillo, E.S., Pixley, F., Stanley, E.R., and Graf, T. (2008). PU.1 and C/EBPalpha/beta convert fibroblasts into macrophage-like cells. Proc. Natl. Acad. Sci. USA *105*, 6057–6062.

Fukuchi, Y., Shibata, F., Ito, M., Goto-Koshino, Y., Sotomaru, Y., Ito, M., Kitamura, T., and Nakajima, H. (2006). Comprehensive analysis of myeloid lineage conversion using mice expressing an inducible form of C/EBP alpha. EMBO J. *25*, 3398–3410.

Funaba, M., and Mathews, L.S. (2000). Identification and characterization of constitutively active Smad2 mutants: evaluation of formation of Smad complex and subcellular distribution. Mol. Endocrinol. *14*, 1583–1591.

Gaarenstroom, T., and Hill, C.S. (2014). TGF-β signaling to chromatin: how Smads regulate transcription during self-renewal and differentiation. Semin. Cell Dev. Biol. *32*, 107–118.

Graf, T. (2011). Historical origins of transdifferentiation and reprogramming. Cell Stem Cell *9*, 504–516.

Hockemeyer, D., Soldner, F., Cook, E.G., Gao, Q., Mitalipova, M., and Jaenisch, R. (2008). A drug-inducible system for direct reprogramming of human somatic cells to pluripotency. Cell Stem Cell *3*, 346–353.

Hong, H., Takahashi, K., Ichisaka, T., Aoi, T., Kanagawa, O., Nakagawa, M., Okita, K., and Yamanaka, S. (2009). Suppression of induced pluripotent stem cell generation by the p53-p21 pathway. Nature *460*, 1132–1135.

Ichida, J.K., Blanchard, J., Lam, K., Son, E.Y., Chung, J.E., Egli, D., Loh, K.M., Carter, A.C., Di Giorgio, F.P., Koszka, K., et al. (2009). A small-molecule inhibitor of tgf-Beta signaling replaces sox2 in reprogramming by inducing nanog. Cell Stem Cell *5*, 491–503.

Ikushima, H., and Miyazono, K. (2010). TGFbeta signalling: a complex web in cancer progression. Nat. Rev. Cancer *10*, 415–424.

Islam, S., Kjällquist, U., Moliner, A., Zajac, P., Fan, J.B., Lönnerberg, P., and Linnarsson, S. (2012). Highly multiplexed and strand-specific single-cell RNA 5′ end sequencing. Nat. Protoc. *7*, 813–828.

Islam, S., Zeisel, A., Joost, S., La Manno, G., Zajac, P., Kasper, M., Lönnerberg, P., and Linnarsson, S. (2014). Quantitative single-cell RNA-seq with unique molecular identifiers. Nat. Methods *11*, 163–166.

Kajimura, S., Seale, P., Kubota, K., Lunsford, E., Frangioni, J.V., Gygi, S.P., and Spiegelman, B.M. (2009). Initiation of myoblast to brown fat switch by a PRDM16-C/EBP-beta transcriptional complex. Nature *460*, 1154–1158.

Kato, Y., Habas, R., Katsuyama, Y., Näär, A.M., and He, X. (2002). A component of the ARC/Mediator complex required for TGF beta/Nodal signalling. Nature *418*, 641–646.

Kim, S.I., Oceguera-Yanez, F., Hirohata, R., Linker, S., Okita, K., Yamada, Y., Yamamoto, T., Yamanaka, S., and Woltjen, K. (2015). KLF4 N-terminal variance modulates induced reprogramming to pluripotency. Stem Cell Reports *4*, 727–743.

Koike-Yusa, H., Li, Y., Tan, E.P., Velasco-Herrera, Mdel.C., and Yusa, K. (2014). Genome-wide recessive genetic screening in mammalian cells with a lentiviral CRISPR-guide RNA library. Nat. Biotechnol. *32*, 267–273.

Kriks, S., Shim, J.W., Piao, J., Ganat, Y.M., Wakeman, D.R., Xie, Z., Carrillo-Reid, L., Auyeung, G., Antonacci, C., Buch, A., et al. (2011). Dopamine neurons derived from human ES cells efficiently engraft in animal models of Parkinson's disease. Nature *480*, 547–551.

Kulessa, H., Frampton, J., and Graf, T. (1995). GATA-1 reprograms avian myelomonocytic cell lines into eosinophils, thromboblasts, and erythroblasts. Genes Dev. *9*, 1250–1262.

Langmead, B., and Salzberg, S.L. (2012). Fast gapped-read alignment with Bowtie 2. Nat. Methods *9*, 357–359.

Li, H., Handsaker, B., Wysoker, A., Fennell, T., Ruan, J., Homer, N., Marth, G., Abecasis, G., and Durbin, R.; 1000 Genome Project Data Processing Subgroup (2009). The Sequence Alignment/Map format and SAMtools. Bioinformatics *25*, 2078–2079.

Li, R., Liang, J., Ni, S., Zhou, T., Qing, X., Li, H., He, W., Chen, J., Li, F., Zhuang, Q., et al. (2010). A mesenchymal-to-epithelial transition initiates and is required for the nuclear reprogramming of mouse fibroblasts. Cell Stem Cell *7*, 51–63.

Love, M.I., Huber, W., and Anders, S. (2014). Moderated estimation of fold change and dispersion for RNA-seq data with DESeq2. Genome Biol. *15*, 550.

Lujan, E., Zunder, E.R., Ng, Y.H., Goronzy, I.N., Nolan, G.P., and Wernig, M. (2015). Early reprogramming regulators identified by prospective isolation and mass cytometry. Nature *521*, 352–356.

Maherali, N., and Hochedlinger, K. (2009). Tgfbeta signal inhibition cooperates in the induction of iPSCs and replaces Sox2 and cMyc. Curr. Biol. *19*, 1718–1723.

Massagué, J. (2012). TGFβ signalling in context. Nat. Rev. Mol. Cell Biol. *13*, 616–630.

Massagué, J., Seoane, J., and Wotton, D. (2005). Smad transcription factors. Genes Dev. *19*, 2783–2810.

McLean, C.Y., Bristor, D., Hiller, M., Clarke, S.L., Schaar, B.T., Lowe, C.B., Wenger, A.M., and Bejerano, G. (2010). GREAT improves functional interpretation of cis-regulatory regions. Nat. Biotechnol. *28*, 495–501.

Morita, S., Kojima, T., and Kitamura, T. (2000). Plat-E: an efficient and stable system for transient packaging of retroviruses. Gene Ther. *7*, 1063–1066.

Mullen, A.C., Orlando, D.A., Newman, J.J., Lovén, J., Kumar, R.M., Bilodeau, S., Reddy, J., Guenther, M.G., DeKoter, R.P., and Young, R.A. (2011). Master transcription factors determine cell-type-specific responses to TGF-β signaling. Cell *147*, 565–576.

O'Malley, J., Skylaki, S., Iwabuchi, K.A., Chantzoura, E., Ruetz, T., Johnsson, A., Tomlinson, S.R., Linnarsson, S., and Kaji, K. (2013). High-resolution analysis with novel cell-surface markers identifies routes to iPS cells. Nature *499*, 88–91.

Okita, K., Matsumura, Y., Sato, Y., Okada, A., Morizane, A., Okamoto, S., Hong, H., Nakagawa, M., Tanabe, K., Tezuka, K., et al. (2011). A more efficient method to generate integration-free human iPS cells. Nat. Methods *8*, 409–412.

Pang, Z.P., Yang, N., Vierbuchen, T., Ostermeier, A., Fuentes, D.R., Yang, T.Q., Citri, A., Sebastiano, V., Marro, S., Südhof, T.C., and Wernig, M. (2011). Induction of human neuronal cells by defined transcription factors. Nature *476*, 220–223.

Pereira, M., Pfisterer, U., Rylander, D., Torper, O., Lau, S., Lundblad, M., Grealish, S., and Parmar, M. (2014). Highly efficient generation of induced neurons from human fibroblasts that survive transplantation into the adult rat brain. Sci. Rep. *4*, 6330.

Pfisterer, U., Wood, J., Nihlberg, K., Hallgren, O., Bjermer, L., Westergren-Thorsson, G., Lindvall, O., and Parmar, M. (2011). Efficient induction of functional neurons from adult human fibroblasts. Cell Cycle *10*, 3311–3316.

Quinlan, A.R., and Hall, I.M. (2010). BEDTools: a flexible suite of utilities for comparing genomic features. Bioinformatics *26*, 841–842.

Ramírez, F., Dündar, F., Diehl, S., Grüning, B.A., and Manke, T. (2014). deepTools: a flexible platform for exploring deep-sequencing data. Nucleic Acids Res. *42*, W187–W191.

Redmer, T., Diecke, S., Grigoryan, T., Quiroga-Negreira, A., Birchmeier, W., and Besser, D. (2011). E-cadherin is crucial for embryonic stem cell pluripotency and can replace OCT4 during somatic cell reprogramming. EMBO Rep. *12*, 720–726.

Rohland, N., and Reich, D. (2012). Cost-effective, high-throughput DNA sequencing libraries for multiplexed target capture. Genome Res. *22*, 939–946.

Ross-Innes, C.S., Stark, R., Teschendorff, A.E., Holmes, K.A., Ali, H.R., Dunning, M.J., Brown, G.D., Gojis, O., Ellis, I.O., Green, A.R., et al. (2012). Differential oestrogen receptor binding is associated with clinical outcome in breast cancer. Nature *481*, 389–393.

Samavarchi-Tehrani, P., Golipour, A., David, L., Sung, H.K., Beyer, T.A., Datti, A., Woltjen, K., Nagy, A., and Wrana, J.L. (2010). Functional genomics reveals a BMP-driven mesenchymal-to-epithelial transition in the initiation of somatic cell reprogramming. Cell Stem Cell *7*, 64–77.

Shi, Y., and Massagué, J. (2003). Mechanisms of TGF-beta signaling from cell membrane to the nucleus. Cell *113*, 685–700.

Shi, Y., Wang, Y.F., Jayaraman, L., Yang, H., Massagué, J., and Pavletich, N.P. (1998). Crystal structure of a Smad MH1 domain bound to DNA: insights on DNA binding in TGF-beta signaling. Cell *94*, 585–594.

Soufi, A., Donahue, G., and Zaret, K.S. (2012). Facilitators and impediments of the pluripotency reprogramming factors' initial engagement with the genome. Cell *151*, 994–1004.

Subramanian, A., Tamayo, P., Mootha, V.K., Mukherjee, S., Ebert, B.L., Gillette, M.A., Paulovich, A., Pomeroy, S.L., Golub, T.R., Lander, E.S., and Mesirov, J.P. (2005). Gene set enrichment analysis: a knowledge-based approach for interpreting genome-wide expression profiles. Proc. Natl. Acad. Sci. USA *102*, 15545–15550.

Takahashi, K., and Yamanaka, S. (2006). Induction of pluripotent stem cells from mouse embryonic and adult fibroblast cultures by defined factors. Cell *126*, 663–676.

Thomas, P.D., Campbell, M.J., Kejariwal, A., Mi, H., Karlak, B., Daverman, R., Diemer, K., Muruganujan, A., and Narechania, A. (2003). PANTHER: a library of protein families and subfamilies indexed by function. Genome Res. *13*, 2129–2141.

Tojo, M., Hamashima, Y., Hanyu, A., Kajimoto, T., Saitoh, M., Miyazono, K., Node, M., and Imamura, T. (2005). The ALK-5 inhibitor A-83-01 inhibits Smad signaling and epithelial-to-mesenchymal transition by transforming growth factor-beta. Cancer Sci. *96*, 791–800.

Tzelepis, K., Koike-Yusa, H., De Braekeleer, E., Li, Y., Metzakopian, E., Dovey, O.M., Mupo, A., Grinkevich, V., Li, M., Mazan, M., et al. (2016). A CRISPR Dropout Screen Identifies Genetic Vulnerabilities and Therapeutic Targets in Acute Myeloid Leukemia. Cell Rep. *17*, 1193–1205.

van Oevelen, C., Collombet, S., Vicent, G., Hoogenkamp, M., Lepoivre, C., Badeaux, A., Bussmann, L., Sardina, J.L., Thieffry, D., Beato, M., et al. (2015). C/EBPα activates pre-existing and de novo macrophage enhancers during induced pre-B cell transdifferentiation and myelopoiesis. Stem Cell Reports *5*, 232–247.

Vierbuchen, T., Ostermeier, A., Pang, Z.P., Kokubu, Y., Südhof, T.C., and Wernig, M. (2010). Direct conversion of fibroblasts to functional neurons by defined factors. Nature *463*, 1035–1041.

Xi, Q., He, W., Zhang, X.H., Le, H.V., and Massagué, J. (2008). Genome-wide impact of the BRG1 SWI/SNF chromatin remodeler on the transforming growth factor beta transcriptional program. J. Biol. Chem. *283*, 1146–1155.

Xie, H., Ye, M., Feng, R., and Graf, T. (2004). Stepwise reprogramming of B cells into macrophages. Cell *117*, 663–676.

Xu, J., Liu, X., Jiang, Y., Chu, L., Hao, H., Liua, Z., Verfaillie, C., Zweier, J., Gupta, K., and Liu, Z. (2008). MAPK/ERK signalling mediates VEGF-induced bone marrow stem cell differentiation into endothelial cell. J. Cell. Mol. Med. *12* (6A), 2395–2406.

Yamakawa, T., Sato, Y., Matsumura, Y., Kobayashi, Y., Kawamura, Y., Goshima, N., Yamanaka, S., and Okita, K. (2016). Screening of human cDNA library reveals two differentiation-related genes, HHEX and HLX, as promoters of early phase reprogramming toward pluripotency. Stem Cells *34*, 2661–2669.

Zhang, Y.E. (2009). Non-Smad pathways in TGF-beta signaling. Cell Res. *19*, 128–139.

Zhang, Y., Liu, T., Meyer, C.A., Eeckhoute, J., Johnson, D.S., Bernstein, B.E., Nusbaum, C., Myers, R.M., Brown, M., Li, W., and Liu, X.S. (2008). Model-based analysis of ChIP-Seq (MACS). Genome Biol. *9*, R137.

STAR★METHODS

KEY RESOURCES TABLE

REAGENT or RESOURCE	SOURCE	IDENTIFIER
Antibodies		
CD19 (1D3)	BD Pharmigen	550992, RRID: AB_398483
MAC-1 (M1/70)	BD Pharmigen	52850, RRID: AB_394491
ICAM1-biotin conjugate	Thermo Fisher	13-0541, RRID: AB_466480
CD44-allophycocyanin (APC) conjugate	Thermo Fisher	17-0441, RRID: AB_469390
E-CADHERIN-eFluor 660,	Thermo Fisher	50-3249-82, RRID: AB_11040003
MEFSK4-biotin conjugate	Miltenyi	130-101-875, RRID: AB_2660622
CD73-Alexa Fluor 647	BD Biosciences	561543, RRID: AB_10896329
CD104-APC	Miltenyi	130-106-924, RRID: AB_2654418
CD47-biotin conjugate	BioLegend	127505, RRID: AB_1134125
Strepdavidin PE-Cy7	Thermo Fisher	25-4317, RRID: AB_10116480
p19ARF	Abcam	Ab80, RRID: AB_306197
E-CADHERIN-eFluor 660	Thermo Fisher	50-3249-82, RRID: AB_11040003
CD44-allophycocyanin (APC) conjugate	Thermo Fisher	17-0441, RRID: AB_469391
pSmad3	Cell Signaling	C25A9, RRID: AB_2193207
pSmad2/3	Santa Cruz Biotechnology	11769, RRID: AB_2193189
Chemicals, Peptides, and Recombinant Proteins		
A83-01, Alk4/5/7 inhibitor	Tocris	#2939
SB431542, Alk4/5/7 inhibitor	Tocris	#1614
FGF2	peprotech	100-18B
heparin	Sigma	1304016
ATCC-formulated F-12K Medium	ATCC	30-2004
N2B27	Stem Cell Sciences	SCS-SF- NB-02
BDNF	R&D systems	P23560
GDNF	R&D systems	P39905
NT3	R&D systems	267-N3-005
db-cAMP	Sigma	D0627
CHIR99021	Axon	1386
Noggin	R&D systems	6057-NG-025
LDN-193189	Axon	1509
Doxycycline hyclate	Sigma	D9891
Ascorbic acid (Vitamin C)	Sigma	PHR1008
Human IL-7	Peprotech	200-07
human colony-stimulating factor 1 (hCSF-1)	Peprotech	300-25
Flt3 ligand	Peprotech	300-19
Stem cell factor	Peprotech	300-07
Deposited Data		
RNA-Seq, ChiP-Seq, and ATAC-Seq data	This study	GEO: GSE85178
Experimental Models: Cell Lines		
TNG MKOS mouse line	Keisuke Kaji lab	N/A
PreB cells from CebpαER mice	Thomas Graf lab	N/A
C2C12	ATCC	CRL-1772
Human fetal lung (hFL1)	ATCC	CCL-153

(*Continued on next page*)

REAGENT or RESOURCE	SOURCE	IDENTIFIER
Continued		
Oligonucleotides		
See Table S3 for guide RNA sequences	This study	N/A
See Table S3 for iPSC ChIP qPCR primers	This study	N/A
See Table S3 for B cell to macrophage transdifferentiation primers	This study	N/A
See Table S3 for ChIP-qPCR primers for C10 pre-B cells	This study	N/A
Recombinant DNA		
pMX-Smad3CA	This study	N/A
pMX-Smad2CA	This study	N/A
pMXs-dsRed	(Hong et al., 2009)	Addgene #22724
pMXs-BFP	This study	N/A
pCXLE-h*Smad2CA*	This study	N/A
pCXLE- h*Smad3CA*	This study	N/A
pCXLE-hOct3/4-shp53-F	(Okita et al., 2011)	Addgene #27077
pCXLE-hSK(Sox2/Klf4)	(Okita et al., 2011)	Addgene #27078
pCXLE-hUL(L-Myc/Lin28)	(Okita et al., 2011)	Addgene #27080
pMIG-CEBPβ	(Di Stefano et al., 2014)	N/A
pMSCV-PRDM16	(Kajimura et al., 2009)	N/A
TET-Ascl1	(Vierbuchen et al., 2010)	Addgene #27150
TET-Brn2	(Vierbuchen et al., 2010)	Addgene #27151
TET-Myt1L	(Vierbuchen et al., 2010)	Addgene #27152
TET-NeuroD1	(Vierbuchen et al., 2010)	Addgene #30129
FUW-M2rtTA	(Hockemeyer et al., 2008)	Addgene #20342
FUW-Smad3CA	This study	N/A
pKLV-U6gRNA(BbsI)-PGKpuro2ABFP	(Koike-Yusa et al., 2014)	N/A
Software and Algorithms		
DESeq2 (Version 1.6.3) package of Bioconductor	Love et al., 2014	N/A
SAMtools	(Li et al., 2009)	N/A
Bowtie 2	(Langmead and Salzberg, 2012)	N/A
BEDTools	(Quinlan and Hall, 2010)	N/A
See also RNA-sequencing and data analysis section	This study	N/A
See also ChIP-seq and ATAC-seq data analysis sections	This study	N/A

CONTACT FOR REAGENT AND RESOURCE SHARING

Further information and requests for resources and reagents should be directed to and will be fulfilled by the Lead Contact, Keisuke Kaji, at keisuke.kaji@ed.ac.uk

EXPERIMENTAL MODEL AND SUBJECT DETAILS

All animal experiments for the iPSC generation were approved by the University of Edinburgh Animal Welfare and Ethical Review Body, performed at the University of Edinburgh, and carried out according to regulations specified by the Home Office and Project License. All experiments for the B-to-M conversion were approved by the Ethics Committee of the Barcelona Biomedical Research Park (PRBB) and performed according to Spanish and European legislation. The use of human fetal fibroblasts is in compliance with Lund University ethical guidelines and practices.

Primary cell lines

The TNG MKOS reprogramming line harbors a targeted *Nanog*-GFP (TNG) reporter at the AUG start site of exon 1 of the *Nanog* locus, thereby creating a reporter of the endogenous promoter activity, while also producing a heterozygous KO of the targeted allele (Chambers et al., 2007). The TNG line was targeted with an *MKOS-ires-mOrange* dox inducible reprogramming cassette that also harbored a CAG promoter driven rtTA, targeted to the *Sp3* locus (Chantzoura et al., 2015). The resulting TNG MKOS ESC line

was used to generate TNG MKOS MEFs from E12.5 chimeric embryos (mixture of male and female embryos) via morula aggregation, and a reprogrammable TNG MKOS mouse line via blastocyst injection. Percentages of Tg MEFs from each chimeric embryo were assessed by mOrange expression after culturing a small portion of the MEFs in the presence of 1 μg/ml doxycycline (dox) for 48 hours before freezing down the rest of the MEFs. The TNG MKOS mouse line was backcrossed with CD1 mice ~3-6 generations before used to generate TNG MKOS MEFs from mixture of male and female E12.5 embryos.

MEFs were cultured at 37°C, 5% CO_2 in MEF medium (Glasgow's Minimum Essential Medium (GMEM), 10% FCS, non-essential amino acids, 2 mM L-Glutamine, 1 mM sodium pyruvate, 100 U/ml penicillin/streptomycin, all from Life technologies) supplemented with 5 ng/ml FGF2 (Peptrotech) and 1 ng/ml heparin (Sigma-Aldrich). The TNG MKOS ESC line was maintained on gelatin in ESC medium (MEF medium without FGF2 and heparin, supplemented with human LIF 100 U/ml).

Pre-B cells (a mixture of both male and female) were isolated from bone marrow of inducible CebpαER mice (Fukuchi et al., 2006) using monoclonal antibodies to CD19 (1D3, BD Pharmigen), using MACS (Miltenyi Biotech).

C2C12 cells (female) were obtained from ATCC and cultured in DMEM supplemented with 10% FBS, L-glutamine and penicillin/streptomycin (Life Technologies).

Human (female) fetal lung (hFL1, ATCC- CCL- 153) fibroblasts were cultured in ATCC-formulated F-12K Medium, supplemented with 10% fetal bovine serum at 37°C, 5% CO_2.

METHOD DETAILS

MEF Reprogramming

Reprogramming was performed on gelatin-coated plates with ESC medium containing doxycycline (dox) (300 ng/ml, Sigma-Aldrich), 10 μg/ml Vitamin C (Sigma-Aldrich), with or without 500 nM A83-01 (A83) (Tocris, #2939) or 10 μM SB431542 (SB43) (Tocris, #1614,). Reprogramming experiments, except immunoprecipitation and ChIP, utilized a mixture of Tg MEF harvested from E12.5 chimeric embryos generated by morula aggregation as described above (5%) and wild-type MEFs (95%) in order to avoid saturating the number of iPSC colonies in the assays. MEFs were defrosted at high confluence (> 70%) in MEF media 2-3 days prior to plating for reprogramming. One day prior to initiating reprogramming, the cells were seeded on gelatin pre-coated plates at $1x10^5$ total cells for 1 well of a 6-well plate, or $7x10^5$ total cells for a 10cm plate, which is $5x10^3$ or $3.6x10^4$ Tg cells, respectively. One day after plating, MEF media is replaced with reprogramming media containing 300 ng/ml dox to activate expression of MKOS reprogramming factors. Reprogramming media is replenished every 2 days until the end of the experiment.

For immunoprecipitation and ChIP, which required a large amount of cells undergoing reprogramming, we used Tg MEFs derived from a TNG MKOS mouse line ($Nanog^{+/GFP}$ and $Sp3^{+/tetO-MKOS-ires-mOrange, CAG-rtTA}$) with 129 and CD1 mixed genetic background and perform the experiments with 100% Tg cells with $5x10^4$/well (6 well plate) or $5x10^5$ cells/10 cm dish.

B cell to macrophage transdifferentiation

Pre-B cells were isolated from bone marrow of inducible CebpαER mice (Fukuchi et al., 2006) using monoclonal antibodies to CD19 (1D3, BD Pharmigen), using MACS (Miltenyi Biotech). After infection with Smad3CA or control retroviral vectors, pre-B cells were plated on S17 feeder cells in RPMI medium supplemented with 10% FBS (Life technologies), 10 ng/mL IL-7 (Peprotech). Transdifferentiation was induced by treating the cells with 1 μM 4-OHT (Sigma), supplemented with 10 ng/mL of IL-7 (Peprotech), IL-3 (Peprotech), human colony-stimulating factor 1 (hCSF-1), FLT3 ligand (Peprotech) and stem cell factor (SCF). Cells stained with antibodies against MAC-1 (44, BD Pharmigen) and CD19 (1D3, BD Pharmigen) were analyzed with a FACS LSR fortessa flow cytometer (BD Biosciences) and FlowJo software (Tree Star, V10). S17 feeder cells were maintained in DMEM supplemented with 10% FBS, L-glutamine and penicillin/streptomycin (Life Technologies).

Myoblast to adipocyte transdifferentiation

C2C12 cells were obtained from ATCC and cultured in DMEM supplemented with 10% FBS, L- glutamine and penicillin/streptomycin (Life Technologies). C2C12 were infected with Cebpβ, Prdm16 and either Smad3CA or control retroviral vectors for 48 hours. After the infection, cells were cultured in adipogenic-induction medium (DMEM containing 10% FBS, 0.5 mM isobutylmethylxanthine, 125 nM indomethacin, 5M dexamethasone, 850 nM insulin, 1 nM T3 and 1 M rosiglitazone (Santa Cruz) for 2 days. Subsequently, medium was switched to DMEM containing 10% FBS, 850 nM insulin, 1 nM T3 and 1 μM rosiglitazone, and cells were maintained for 4 days in this culture condition before Red Oil O staining was performed. All the reagents were purchased from Sigma unless otherwise indicated. For Oil Red O staining, cultured cells were washed with PBS and fixed in formalin for 10 min at room temperature. Cells were incubated for 30 min at room temperature with 60% filtered Oil Red O stock solution (0.3 g/100 mL of isopropanol) (Sigma). Cells were washed with 60% isopropanol and then water before visualization. Lipid levels were quantified by extracting Oil Red O stained cells with isopropanol and measuring absorbance at 510 nm.

Generation of human iNs

Human fetal lung (hFL1, ATCC- CCL- 153) fibroblasts were purchased from the American Type Culture Collection (ATCC) and were expanded in MEF medium and grown at 37°C in 5% CO_2. hFL1 were converted to hiNs by transduction of the cells with lentiviral vectors (LVs) encoding for the conversion factors Ascl1, Brn2a, Myt1L, NeuroD1 with or without Smad3CA (MOI = 5) and co- transduction of the transactivator (FUW-rtTA-SM2, Addgene) (MOI = 10). Transgene expression was initiated by doxycycline (2 μg/ mL,

Saveen & Werner) administration on day 5 after infection. On day 3 of transgene expression, cells received neuronal induction medium (N2B27 + doxycycline 2 μg/ mL) supplemented with CHIR99201 (2 μM, Axon), noggin (100 ng/ mL, R&D systems), LDN (0.5 μM, Axon), LM4A22 (2 ng/ mL, R&D system), GDNF (2 ng/ mL, R&D system), NT3 (10 ng/ mL, R&D Systems) and db-cAMP (0.5 mM, Sigma). For immunocytochemistry with mouse anti-MAP2 antibodies (1:500, Sigma), hiNs were fixed in PFA (4%, 15 min at room temperature) 7, 12 and 23 days after transgene activation. Neuronal conversion efficiency was assessed by quantification of DAPI+/ MAP2+ hiNs using Cellomics Array Scan (Array Scan VTI, Thermo Fischer). Using the program "Target activation," 20 fields (10x magnification) were automatically captured in a spiral fashion (from center to outside) and conversion efficiency was determined as the ratio of total number of hiNs present at time of analysis and the number of input fibroblasts.

For RNA analysis, hiNs were detached using Accutase at days 12 and 23 after transgene expression and after gentle trituration, filtered through cell strainer caps in order to obtain a single cell suspension. Cells were spun down and resuspended in the staining buffer (HBSS supplemented with 45% BSA) for blocking (15 min at room temperature) before staining with hNCAM-APC antibody (1:50) for 15 minutes at room temperature. A final centrifugation step and resuspension in HBSS 45% BSA, supplemented with the secondary antibody, Nuclear Orange (ATT Bioquest) and DNase, were perfomed and viable, $NCAM^+$ cells were isolated by flow cytometry for RNA-seq library preparation.

For electrophysiological recordings, hFL1s were converted to iN on glass coverslips. Total 3 coverslips each on 3 different days, from 2 independent cell conversion experiments were used. Patch-clamp electrophysiology was performed on hiNs at day 23-25 post-conversion. Cultured hiNs were grown on coverslips and transferred to a recording chamber and submerged in a continuously flowing Krebs solution gassed with 95% O_2 - 5% CO_2 at 28°C. The composition of the standard solution was: 119 mM NaCl, 2.5 mM KCl, 1.3 mM $MgSO_4$, 2.5 mM $CaCl_2$, 25 mM Glucose and 26 mM $NaHCO_3$. Recordings were made with a Multiclamp 700B amplifier (Molecular Devices), using borosilicate glass pipettes (3–7 MOhm) filled with the following intracellular solution: 122.5 mM potassium gluconate, 12.5 mM KCl, 0.2 mM EGTA, 10 mM HEPES, 2 mM MgATP, 0.3 mM Na_3GTP and 8 mM NaCl adjusted to pH 7.3 with KOH. Data were acquired with pClamp 10.2 (Molecular Devices); current was filtered at 0.1 kHz and digitized at 2 kHz. Cells with neuronal morphology with round cell body were selected for whole-cell patch clamp. Resting membrane potentials were monitored immediately after breaking-in in current-clamp mode. Thereafter, cells were kept at a membrane potential of −60 mV to −80 mV, and 500 ms currents were injected from −20 pA to +90 pA with 10 pA increments to induce action potentials. For sodium and potassium current measurements cells were clamped at −70mV and voltage-depolarizing steps were delivered for 100 ms at 10 mV increments.

Western Blotting

For pSmad3 blots, to confirm TGF-β inhibitors performed as expected, cells were serum starved for 1 hour in GMEM then placed in fibroblast media containing 10 ng/ml TGF-β (R&D Systems) with or without TGF-β inhibitors, then western blotting was performed as for all experiments, as follows. Cells were harvested in Trypsin-EDTA, lysed with 1x Nupage LDS lysis buffer (Life Technologies), with or without phosphatase inhibitors (HALT, Life Technologies), heated to 95°C for 10 minutes followed by sonication of 3 cycles of 15 s on the Misonix XL2000 sonicator on setting 2. Protein concentration was measured by BCA assay (Life Technologies), and 1-100 μg lysate was mixed with DTT (final 100 μM), then run at 150 V and transferred at 50 V for 3-hours with BioRad Mini-PROTEAN and Mini Trans-Blot Cell tanks respectively. Antibodies used included pSMAD2/3 antibody (Santa Cruz sc-11769), and B-ACTIN-HRP antibody (Abcam, ab20272l).

Plasmids

For *Smad2CA/3CA* expression constructs, PCR primers were designed to amplify *Smad2*, *Smad3* cDNA with base pair mismatches at the 3′ end, to exchange serine codons for glutamic acid (SSMS → SEME) in *Smad2* or aspartic acid (SSVS → DDVD) in *Smad3* in the C terminus of the proteins (Chipuk et al., 2002; Funaba and Mathews, 2000). The primers used for mouse *Smad2CA* were forward-CTAGGGTAGATTTACCGGGC, Reverse-CGAGTCTTTGATGGGTTTACTCCATCTCTGAGCATCGCACTGAA, and for *Smad3CA* forward- GCTGGCGCCGGAACCAATTCAGTCGACGTGACCCTTCGGTGCCAG, reverse-CTAATCCACATCGTCACAGCGGATGCT CGGGGAACCCATCTGGGT. The PCR products were then cloned into the TOPO Blunt vector and subsequently cloned into pMXs or FUW expression vectors. A control vector pMXs-BFP was generated by inserting *EBFP2* cDNA in the pMXs-gw (Addgene #18656). pCXLE-h*Smad2CA* and pCXLE- h*Smad3CA* were generated by inserting human *Smad2CA* and *Smad3CA* coding sequence into pCXLE-gw (Addgene #37626). Other pCXLE vectors were obtained from Addgene (Okita et al., 2011). The *Cebpβ* expression vector has been described previously (Di Stefano et al., 2014). The *PRDM16* expression vector was kindly provided by Dr Kajimura (Kajimura et al., 2009). The lentiviral vectors used for hiN generation were described before (Pang et al., 2011; Pfisterer et al., 2011; Vierbuchen et al., 2010). Plasmids used in this work are listed in the Key Resource Table.

Fluorescence activated cell sorting

Preparation of cells for sorting was performed in PBS with 2% FCS at 4°C. The following antibodies were used with indicated dilution: ICAM1-biotin conjugate (1:100, eBioscience, #13-0541), CD44-allophycocyanin (APC) conjugate (1:300, eBioscience, #17-0441), E-CADHERIN-eFluor 660 (1:300, eBioscience, #50-3249-82), MEFSK4-biotin conjugate (1:100, Miltenyi, 130-101-875), CD47-biotin conjugate (1:100, BioLegend, 127505), CD73-Alexa Fluor 647 (1:300, BD Biosciences, 561543) and CD104-APC (1:300, Miltenyi, 130-106-924) and Strepdavidin PE-Cy7 (1:1500, eBioscience, #25-4317).

Immunofluorescence microscopy

Cells were reprogrammed in 6-well plates on 18 mm circular coverslips (Fisher Scientific) for confocal imaging, or directly on the plastic for whole well imaging, both coated with gelatin. Cells were fixed with 4% Paraformaldehyde for 10 minutes, permeabilized in 0.1% Triton-X in PBS for 45 minutes, blocked in 5% normal goat serum in PBS for 1 hour at room temperature, and then stained in blocking solution with primary antibody overnight at 4°C. The next day, secondary antibody was applied in blocking solution for 1 hour at room temperature. Slides where then mounted with prolong gold with or without DAPI (Life technologies). The following antibodies were used with indicated dilution: p19ARF (1:300, Abcam, Ab80), E-CADHERIN-eFluor 660 (1:300, eBioscience, #50-3249-82), CD44-allophycocyanin (APC) conjugate (1:300, eBioscience, #17-0441), pSmad3 (1:100, Cell Signaling, C25A9). For confocal microscopy, all imaging was performed with a Leica TSC SP2 and processed using Adobe Photoshop. For whole well colony and cell counting, the sistched whole well images were taken and analyzed using the *Celigo* S Cell Cytometer (Nexcelom).

Smad2/3 double KO MEF reprogramming

An *EF1α* promoter-driven *Cas9* expression cassette was targeted into the *Rosa26* locus of TNG MKOS ES cell line, which has a doxycycline (dox)-inducible *MKOS-ires-mOrange* reprogramming and a CAG promoter-driven *rtTA* expression cassette in the *Sp3* locus and a *Nanog*-GFP reporter (Chantzoura et al., 2015). The resulting Cas9 TNG MKOS ES cells were used to generate chimeric embryos via morula aggregation. Each E12.5 embryo was dissociated with trypsin/EDTA and 1/20 and 19/20 of the cells were seeded in one well of a 12-well plate in the presence of dox and a 10 cm dish in the absence of dox, respectively. Two days later, the cells in the 12-well plate were used to determine contribution of the Tg cell by measuring percentage of mOrange$^+$ cells with flow cytometry. MEFs with high (> 90%) Tg cell contribution were harvested from the 10 cm dishes, pooled and cryopreserved at 5x10^6 cells/vial as passage 0. Lentiviral gRNA expression vectors were constructed by inserting double strand oligos encoding the following gRNA sequencing into pKLV-U6gRNA(BbsI)-PGKpuro2ABFP. Defrosted cells were seeded into a T25 flask/vial (passage 1) and became confluent within 2 days. The MEFs were harvested and seeded into a 6-well plate at 150,000 cells/well to make samples for western blotting. A portion of remaining MEFs were mixed with wild-type (WT) MEFs (129 strain) as the percentage of the Tg MEFs became 5%, and the same amount (150,000 cells/well) of the mixed MEFs were seeded in a 6 well plate for reprogramming experiments (passage 2). 24 hours later, cells for both experiments were infected with the same amount of gRNA viruses (MOI3) for 4 hours. For western blotting, cells were harvested 72 hours after infection, and 3 μg of cell lysate was used to detect SMAD2/3 (1:1000, Cell Signaling, #5678) and β-ACTIN (1:2500, Abcam, #ab20272). For reprogramming, dox was administrated 24 hours infection, and number of *Nanog*-GFP$^+$ colonies was assessed on day 14 of reprogramming. gRNA sequences are in Table S3.

Retroviral mediated gene transduction for iPSC reprogramming enhancement

Ten micrograms of pMXs retroviral vector with *BFP*, *dsRed* (Addgene, 22724) (Hong et al., 2009), *Smad2CA* or *Smad3CA*, were transfected into 1.7x10^6 Plat-E packaging cells in a 10 cm dish (Morita et al., 2000), in 8 mL of ES media, using the $CaCl_2$-HBS transfection method. The cells were cultured for 24 hours at 32°C, and the viral supernatant was collected. Filtrated supernatant with 0.45 μm Whatman acetate filters was then mixed with 8 μg/ml polybrene, of which 2 mL was added to each well containing 1x10^5 MEFs. After 6-hours of infection at 32°C, media was changed to initiate reprogramming and cells were placed back at 37°C.

Human fibroblast reprogramming to iPSCs

Commercially available neonatal human dermal fibroblasts (nHDFs, Life technologies) were cultured as per manufacturer's instructions. Reprogramming was accomplished by transfection of episomal plasmids as previously published (Okita et al., 2011). Briefly 5x10^5 passage 4 nHDFs were nucleofected (Amaxa, Human Dermal Fibroblast Nucleofector Kit, program U023) with 5 μg total plasmids containing equal amounts pCXLE-hOct3/4-shp53 with pCXLE-hSK and pCXLE-hUL, +/− pCXLE-Smad3CA or pCXLE-Smad2CA. After 5 days of expansion in nHDF media, cells were lifted with EDTA and 1.9x10^5 were plated on matrigel coated plates, media was then changed 2 days later to E8 media (Life Technologies) supplemented with pen/strep (100 U/ml) and exchanged every 2 days until cells reached ~70% confluence, at which point media was changed daily. Number of NANOG$^+$ colonies was assessed on day 21 of reprogramming immunofluorescence with anti-human NANOG antibody (1:200, BD PharMingen, #560482).

RNA-sequencing and data analysis

RNA-seq was performed with the single-cell tagged reverse transcription (STRT) platform using 500 cells/sample, which had been snap frozen in PBS with RNase inhibitor, then processed as previously described (Islam et al., 2012; Islam et al., 2014). The RNA-seq reads were processed by an automated pipeline as outlined in a recent publication (Islam et al., 2014). Counts data, based on both read and molecule counts, were processed with the DESeq2 (Version 1.6.3) package of Bioconductor(Love et al., 2014). However we found that the processing steps used to produce molecule count data lead to the loss of read counts for low-abundance genes, so proceeded with analysis on read count data, analogous to standard RNA-seq. 2-3 technical replicates per sample tightly clustered and therefore summed across columns prior to further data processing. As per the standard DESeq2 protocol, normalized expression estimates were obtained by adjusting columns by a size factor corresponding to library size. For read counts, for example, this adjustment ranged from 0.57 to 0.71. Data were transformed to log2 scale by use of DESeq2's rlog() command, which also minimizes differences at very low count levels. The clustering patterns of genes in the two series were assessed based on a matrix of the mean of biological replicate samples. Two matrices were constructed containing the Cont and +SmCA3 series. The mean of MEF and ES samples were included in both series. To reduce noise, genes with background level expression (no mean count > = 2) or low variation

(coefficient of variation < 100) in both series were removed from this matrix. The matrix was clustered by use of the clara() function in R, which approximates the Partitioning Around Medoids algorithm. A K value of 8 clusters was chosen as 7 out of 8 clustered patterns were similar between the Cont and +SmCA3 series. Mean normalized counts of each cross-classified gene group (with > 100 genes) identified in the chord diagram were shown. Gene ontology statistical overrepresentation test was performed using PANTHER (http://pantherdb.org/) with GO-Slim Biological Process with (Figure S4J) and without the Bonferroni correction for multiple testing (Figures 7I and 7K) (Thomas et al., 2003).

Co-immunoprecipitation

Cells were suspended in hypotonic solution (10 mM HEPES pH7.9, 1.5 mM $MgCl_2$, 10 mM KCl) containing proteinase inhibitors (cOmplete protease inhibitor cocktail, Roche) and phosphatase inhibitors (HALT, Life Technologies) with 0.5% NP40, briefly vortexed and left on ice for 5 minutes prior to centrifugation at 500 g to pellet nuclei. The pellet was suspended in high salt solution (HEPES pH 7.9, 1.5 mM $MgCl_2$, 0.2 mM EDTA, 400 mM NaCl, 25% Glycerol) to extract nuclear protein. The corrected nuclear lysates were pre-cleared in IP-buffer (20 mM HEPES, 1.5 mM $MgCl_2$, 0.2 mM EDTA, 20% Glycerol, 100mM NaCl, protease and phosphates inhibitors) overnight at 4°C with 25 μL Dynabeads-Protein G (Life Technologies, #10003D) that were pre-blocked blocked in 2% milk with no-salt buffer (20 mM HEPES pH 7.9, 1.5 mM $MgCl_2$, 0.2 mM EDTA, 20% Glycerol, phosphatase and protease inhibitors). Bead-antibody complexes were generated by incubating 2 μg of antibody or control IgG with 25 μL blocked beads in IP buffer rotating overnight at 4°C. Antibody bound beads were then magnetically isolated and added to 200 μg pre-cleared lysate for a 3-hour incubation, rotating at 4°C. Bead-protein complexes were then washed 3 times in IP buffer and processed for western in LDS buffer as describe above. Antibodies used for co-IP experiments included Rb-SMAD3 (Abcam, #ab28379), ms-SMAD3 (Abcam, #AF9F7), OCT4 (Santa Cruz Biotechnology, #sc8628), SOX2 (Santa Cruz Biotechnology, #sc17320), KLF4 (Santa Cruz Biotechnology, #sc20691), DPY30 (Sant Cruz Biotechnology, #sc167677), p300 (Thermo Scientific, #MS-586-PO), BRG1 (Santa Cruz Biotechnology, #sc17796), MED15 (Sigma Aldrich, SAB2500761), and IgG (Millipore).

Chromatin Immunoprecipitation followed by high-throughput DNA sequencing (ChIP-seq)

Fixation

Cells were grown to 80% confluency and fixed by addition of formaldehyde (final 1%) for 12 minutes with gentle shaking at room temperature. Formaldehyde was quenched with glycine (final 0.125 M) for 5 minutes shaking at room temp. Samples where then washed once with cold PBS containing protease inhibitors (cOmplete protease inhibitor cocktail, Roche) collected with silicon scrapers and centrifuged at 300 g for 5 minutes. Cells were re-suspended in PBS with protease inhibitor, and ~$2x10^6$ cells (based on sacrificed plate cell counts) were aliquoted into 1.5 mL tubes, centrifuged at 500 g and pellets were snap frozen on dry ice.

Nuclear isolation and sonication

A pellet of ~$2x10^6$ cells was thawed and nuclei were isolated by re-suspension in lysis buffer (5 mM Pipes pH 8.0, 85 mM KCl, 1% NP40) with fresh protease inhibitors (cOmplete protease inhibitor cocktail, Roche) for 20 minutes on ice followed by brief vortexing and centrifugation at 500 g for 10 minutes at 4°C. The nuclear pellet was re-suspended in 300 ul IP buffer (0.5% SDS, 1% Triton, 2 mM EDTA, 20 mM Tris-HCl pH 8.0, 150 mM NaCl, fresh protease inhibitors) and sonicated to produce ~200-300 bp fragments in a BioRuptor Sonicator with a total of 5-8 cycles of: 10 pulses of sonication on the "high" setting, each followed by 30 s off. Thus, each sample received between 50-80 pulses of 30 s 'high' sonication. Samples were then diluted in IP buffer without SDS (to final 0.1% SDS), then 8 μg antibody was added for overnight incubation, rotating at 4°C.

Immunoprecipitation and DNA isolation

Dynabeads-ProteinG were blocked for 1-hour in 0.5% BSA in IP buffer with protease inhibitors, prior to incubation with the sonicated antibody bound chromatin suspensions for 4-hours rotating at 4°C. Bead-chromatin complexes were then serially washed for 5 minutes each with the following solutions: IP buffer (150 mM NaCl), followed by IP buffer with high salt concentration (500 mM NaCl), then 1 wash with washing-buffer (10 mM Tris-HCl pH 8.0, 0.25 M LiCl, 0.5% NP40, 0.5% Na-Deoxycholate, 1 mM EDTA), followed by 2 washes with TE (10 mM Tris, 1 mM EDTA). The DNA-protein complex was then eluted from beads by incubation with 100 μl of elution buffer (1% SDS, 10 mM EDTA, 50 mM Tris-HCl pH 8.0) for 30 minutes at 65°C, vortexing every 10 minutes, followed by magnet extraction of beads. The beads were re-washed with 150 μl TE with 1% SDS, magnet extracted, and the TE with remaining DNA solution was added to the eluted samples, followed by 65°C overnight incubation to reverse crosslink the DNA-protein complexes. The dissociated DNA and protein solution was then treated with 4 units proteinase K (NEB) at 37°C for 2 hours. DNA was isolated with SeraMag beads, using 450 μl SeraMag bead solution and 225 μl of 30% PEG in 1.25 M NaCl (Sera-Mag DNA isolation methods below) (Rohland and Reich, 2012). After bead purification, DNA was re-suspended in 30 μl DNase and RNase free H_2O and stored at −80°C prior to library preparation.

ChIP library preparation for sequencing

Blunt end repair

The ChIP'd DNA from ~$2x10^6$ cells, ~6 ng total on average, was prepared in the following reaction mix: 5 μl T4 DNA ligase buffer (NEB), 2 mL 10 mM dNTP's, 0.5 μl end repair mix (0.72 U T4 DNA polymerase, 0.24 U Klenow Fragment, 2.4 U T4 DNA Polynucleotide Kinase), up to 50 μl with ddH2O (DNase/RNase free). Samples were incubated for 30 minutes at 20°C, and then purified with addition

of 50ul SeraMag bead solution and 50 μl 30% PEG solution (in 1.25 M NaCl) as per bead protocol outlined below. The final bead:DNA complexes were re-suspended in 18 μl TE, and 16.5 μl were transferred to new tube.

Adding A bases to 3′ end of DNA fragment

The 16.5 μl blunt end repaired DNA fragments were mixed in the following reaction: 2 μl 10X NEB Buffer 2, 1 μl 4 mM dATP, 0.5 μl Klenow 3′ to 5′ exonuclease minus (NEB). Reaction was incubated at 37°C for 30 minutes.

Adaptor ligation

The 20 μl A-tailed DNA was added to the following mix: 25 μl Quick Ligase Buffer, 1 μl Annealed TruSeq Adaptors (note below), 1.5 μl Quick Ligase (2,000 U/μl NEB) 2.5 μl H_2O. The reaction was incubated 20 minutes at room temperature and then 5 μl of 0.5 M EDTA, pH 8.0. The adaptor ligated DNA fragments were then purified with addition of 50 μl SeraMag bead mix and 50 μl 30% PEG (in 1.25 M NaCl) as per bead protocol below. The final bead:DNA mixture was re-suspended in 15.5 μl TE, and 14 μl was taken to a new tube. TruSeq adaptor annealing was performed by re-suspending adaptors at 100 μM (in 10 mM Tris-HCl pH7.8, 0.1 mM EDTA pH 8.0, 50 mM NaCl), mixing 1:1 with the universal adaptor (100 μM), and annealed using a program of: 2 min at 95°C, 70 cycles of 30 s (95°C decreasing by 1°C each cycle), hold at 4°C. Annealed adaptors were diluted 1:200 (0.25 μM final) and then used for ligation or stored at −20°C and used for up to 5 freeze/thaw cycles.

Library pre-amplification

The adaptor ligated DNA was amplified using the following protocol: 14 μl Adaptor-ligated DNA, 1 μl TruSeq primer cocktail (0.25 μM), 15 μl 2X Kapa HiFi HotStart ready mix. The Libraries were amplified with the following protocol: 45 s at 98°C, 5 cycles of (15 s at 98°C, 30 s at 63°C, 30 s at 72°C), 1 minute of 72°C, hold at 4°C.

Library size selection

After pre-amplification of the library, a size selection step was performed to eliminate free adaptors in solution, and ensure amplification of only the desired size ChIP DNA fragments (300-500bp). First, the volume of DNA solution from pre-amplification step was adjusted to have 50 μl total (added 20 ul ddH_2O). Then, 0.9X (45 μl) beads were added. After 15 minute incubation and 10 minutes magnet extraction, the supernatant (92 μl) was transferred to new tube and beads discarded (containing fragments over ~500 bp). Then 10μl beads (~0.2X) were added to the solution, incubated 15 minutes followed by 10 minute magnet extraction of beads, 2 times 80% EtOH washes, and re-suspension in 11.5 μl TE. Then 10 μl pre-amplified and size selected DNA was transferred to a new tube. From the final library, 1 μl was used to identify the number of additional cycles required, and then the remaining 9 μl were amplified an additional ~11-13 cycles as required. The final library was amplified as follows: 9 μl DNA, 1 μl TruSeq primer cocktail, 20 μl 2X Kapa HiFi HotStart ready mix, and 10 μl H_2O. The library was amplified with a program of 45 s at 98°C, 11-13 cycles of (15 s 98°C, 30 s 63°C, 30 s 72°C), 1-minute 72°C, hold at 4°C. Therefore, each library was amplified a total of 16-18 PCR cycles. After amplification, the DNA was isolated by bead purification by adding 60 μl SeraMag beads following described protocol below. Libraries were mixed at equal concentration and sequenced with Edinburgh Genomics on the Illumina HiSeq 2500 platform.

Determining ChIP final library amplification cycle number required

We took 1 μl of pre-amplified, size-selected DNA, and performed a quantitative PCR reaction as follows: 1 μl library, 2 μl SybrGreen fluorophore (10X), 1 μl TruSeq PCR primer cocktail, 10 μl 2X Kapa HiFi HotStart ready mix, split into 2 wells for qPCR analysis on a lightcycler 480. Amplification conditions were: 3 minutes at 95°C, 20 cycles of (30 s at 95°C, 30 s at 63°C, 30 s at 72°C). Upon identifying the cycle number required to reach 50% amplification, we subtracted 2 from that number (based on optimization experience), and used that as the number of cycles required for the rest of the library.

SeraMag bead isolation of DNA from solution

We produce SeraMag beads solution for DNA extraction in house, based on (Rohland and Reich, 2012). The final bead solution is as follows: 0.1% SeraMag Magnetic Speed-beads (FisherSci, cat.#: 09-981-123), 18% PEG-800 (w/v) (Sigma Aldrich cat.#: 89510), 1M NaCl, 10 mM Tris-HCl pH 8.0, 1 mM EDTA, 0.05% Tween 20. The beads are stored at 4°C, and their efficiency was tested using the Fermentas ladder (Fisher #FERSM1211).

For DNA isolation, the concentration of PEG can be adjusted by adding different volumes of the above SeraMag bead solution to DNA, creating different stringency criteria for DNA-bead interaction, allowing for size selection of DNA fragments (Rohland and Reich, 2012). For example, adding the SeraMag bead solution to a DNA containing solution at 1.2X (ie. 60 μl SeraMag bead mix to 50 μl DNA), for a final concentration of 9.8% PEG, only DNA fragments greater than 100 bp in size bind to the beads. Increasing the ratio of beads to DNA solution (resulting in increased PEG concentration) results in smaller sized fragments binding the beads.

The general protocol for bead mediated DNA purification is to add the SeraMag bead solution directly to the DNA solution, mix well, and let stand for 15 minutes. Then the DNA bound beads are magnet extracted for 5-10 minutes (depending on PEG concentration), at which point the PEG solution is removed leaving ~5 μl in bottom of tube so as not to lose any sample, prior to 2 washes with 80% EtOH with tubes on magnet, air-dried for 2 minutes, and then re-suspended in TE (10 mM Tris-HCl pH 8.0, 0.1 mM EDTA) at desired final volume plus 1.5 μl. After 2-minute incubation, the DNA solution is transferred to a new tube, leaving 1.5 μl remaining so as not to take any beads.

ChIP-seq data analyses

Reads were quality-checked using FastQC and trimmed using Trimmomatic to remove adapters and low quality bases (Bolger et al., 2014). Reads were aligned to the mm10 assembly of the mouse genome using Bowtie 2 with the very-sensitive option (Langmead and Salzberg, 2012). Duplicate reads were removed using Picard Tool's MarkDuplicates command and were filtered for MAPQ > = 40

using SAMtools (Li et al., 2009). Reads aligned to ENCODE blacklist regions were removed using BEDTools (Quinlan and Hall, 2010). Peaks were called using MACS 2 on both individual and merged sample replicates against merged control replicates (Zhang et al., 2008). For narrow peak factors, such as OCT4, peaks were called with the -q 0.01 and —call-summits specified. For broad peak factors, such as SMAD3, peaks were called with the–broad and–broad-cutoff 0.1 options specified. Differentially-bound sites were identified using the DiffBind software package from the Bioconductor project (Ross-Innes et al., 2012). A FDR $\leq$ 0.1 and absolute FC > = 1 were used as the significance threshold. Merged control replicates were used for contrast against each individual sample replicate. The deepTools suite was used to generate normalized input-subtracted read coverage and heatmap scores using the bamCompare and plotHeatmap commands, respectively. Normalized input-subtracted read coverage was generated from merged sample and control replicates with the–ratio subtract and–normalizeTo1x 2150570000 option specified. Heatmaps were generated using the normalized input-subtracted read coverage and peaks called from the merged sample replicates.

ATAC-seq

ATAC-seq was performed according to the published protocol (Buenrostro et al., 2013). For library preparation, 50,000 cells were flow-sorted into PBS, and then nuclei were isolated by re-suspending the cells in lysis buffer (10 mM Tris-HCl pH 7.4, 10 mM NaCl, 3 mM $MgCl_2$, 0.1% NP-40) for 15 minutes on ice, followed by centrifugation at 500 g for 10 minutes at 4°C. Supernatant was removed and nuclei were tagmented by re-suspension in the Nextera transposase mix (22.5 μL ddH2O, 25 μL 2x Tagment DNA buffer, 2.5 μl Tagment DNA enyme) (Nextera DNA sample prep kit, FC-121-1030 and FC-121-1011) and incubated at 37°C for 30 minutes. The tagmentation reactions were terminated and cleaned-up using QIAGEN Minelute enzyme cleanup kit, eluting in a final volume of 10 μL ddH_2O. After tagmentation and cleanup, the DNA was then PCR amplified using Nextera primers, indexes and polymerase mix according to the Nextera DNA sample prep kit protocol. The number of PCR cycles required was determined as follows: the full library was amplified for five cycles, after which a 3 μl aliquot of the 30ul PCR reaction was removed and added to 6 μl of the complete PCR cocktail with addition of 1 μL SybrGreen fluorophore (10,000x). The PCR optimization reaction was run for 20 cycles on the LightCycler480 instrument (same program as master library) to determine the additional number of cycles needed for the remaining 27 μL reactions. The final cycle number was chosen to ensure the PCR was in linear phase growth and had not hit the plateau phase, thus minimizing PCR biases. On average, libraries were produced with ~16 PCR cycles total. To the final 30 μl amplified library, 20 μl ddH_2O was added prior to SeraMag bead cleanup. A ratio of 1.2X Bead:PCR reaction was used to isolate library fragments larger than 100 bp, thus leaving the PCR primers/adaptors out of the final solution sent for sequencing. ATAC libraries were sequenced with Edinburgh Genomics on the Illumina HiSeq 2500 platform.

ATAC-seq data analysis

Reads were quality-checked using FastQC and trimmed using Trimmomatic to remove adapters and low quality bases (Bolger et al., 2014). Reads were aligned to the mm10 assembly of the mouse genome using Bowtie 2 (Langmead and Salzberg, 2012) with the–very-sensitive and -X 2000 options specified. Duplicate reads were removed using Picard Tool's MarkDuplicates command and were filtered using SAMtools for MAPQ > = 30 and the properly-paired (0x2) flag (Li et al., 2009). Reads aligned to the ENCODE blacklist and mitochondrial blacklist regions were also removed using BEDTools (Quinlan and Hall, 2010). The deepTools suite was used to generate normalized read coverage and heatmap scores using the bamCoverage and plotHeatmap commands, respectively (Ramírez et al., 2014). Normalized read coverage was generated from merged sample replicates with the–normalizeTo1x 2150570000 option specified. Heatmaps were generated using the normalized read coverage and peaks called from the merged sample replicates. The data of one of the ATAC-seq replicates had low read counts and demonstrated abnormal features as assessed by PCA and hierarchical clustering, clustering independent of all other samples including the starting MEF population and resulting iPSC cells, and was therefore removed from the analysis.

ChIP-qPCR for reprogramming to iPSCs

The ChIPed DNA was treated with 2 μL RNase (Life Technologies) at 37°C for 20 minutes, and then stored at −20°C. Each ChIP-qPCR reaction used 0.25-0.5 μL of DNA suspension per reaction. The qPCR was normalized to regions upstream of *Oct4* and *Nanog,* which are devoid of SMAD3 or OCT4 binding based on previously published works (Chen et al., 2008; Mullen et al., 2011). Primers are listed in Table S3.

qPCR in B cell to macrophage transdifferentiation

Total RNA was isolated using RNeasy Mini Kit (QIAGEN) according to manufacture's instruction. Double strand complementary DNA (cDNA) was synthesized using the High Capacity RNA-to-cDNA kit (Applied Biosystems). Real-time Polymerase Chain Reaction (PCR) was performed on ViiATM 7 system using Power SYBR® Green PCR Master Mix (Applied Biosystems). The relative gene-expression levels were calculated by 2ΔΔCt method. Primers are listed in Table S3.

Bioinformatics analysis of B cell to macrophage transdifferentiation ChIP-seq data

All sequencing data were mapped onto the mouse genome assembly mm10 (Ensembl GRCm38.78) using STAR, then analyzed with R (3.1.0) using packages from the bioconductor suite (v3.0). Cebpα ChIP-seq data were from GSE71218 (Di Stefano et al., 2016) and GSE53362 (van Oevelen et al., 2015). Peak calling was performed using macs2 (2.1.0.20140616) with default parameters and a threshold of qValue < 1e-2. Motif analyses were performed using sequences 500bp around Cebpα peak summits with RSAT

peaks-motif (defaut parameters). Smad-binding motifs enrichment in Cebpα ChIP-seq was analyzed with matrixScan using a threshold of pValue < 1e-3 and the best hits for each peak were reported. We used as background a Marlov model of order1. Gene ontology enrichments were analyzed using GREAT (McLean et al., 2010).

ChIP-qPCR with C10 pre-B cell line

C10 cells were cultured in PRMI medium (Life Technologies) complemented with 10% FBS (Life Technologies) and were induced to transdifferentiate into macrophage (treatment with 100 nM β-estradiol and grown in medium in presence with 10 ng/mL of Il-3 and CSF-1) as described (Bussmann et al., 2009). 18 hours after Cebpα expression vector infection, cells were cross-linked with 1% formaldehyde for 10 minutes, and then lysed in ChIP buffer composed by mixing SDS buffer (NaCl 100 mM, Tris-Cl pH 8.1 50 mM, EDTA pH 8.0 5 mM, NaN_3 0.2%, SDS 0.5%) and Triton Dilution Buffer (Tris-Cl pH 8.6 100 mM, NaCl 100 mM, EDTA 5 mM, NaN3 0.2% and Triton X-100 5%) at the ratio 2:1. Sonication was performed in a Bioruptor (Diagenode) and soluble material containing fragmented chromatin was then incubated overnight with anti-Smad3 (Abcam, #ab28379) or IgG control antibodies respectively. Immunoprecipitates were recovered with 60 μL of Dynabeads® A or G (Life Technology) and washed 3 times with low salt buffer (HEPES pH 7.5, 50 mM, NaCl 140 mM, Triton X-100 1%), once with high salt buffer (HEPES pH 7.5, 50 mM, NaCl 500 mM, Triton X-100 1%) and decross-linked in freshly prepared elution buffer (1% SDS, $NaHCO_3$ 100 mM and NaCl 500 mM) overnight at 65°C on a shaker. Genomic DNA was eluted using PCR purification kit (QIAGEN). Primers are listed in Table S3.

QUANTIFICATION AND STATISTICAL ANALYSES

A one or two-sided t test was performed using the GraphPad Prism software for Figures 1, 2, 4, and 5. One-sided tests were chosen for experiments where we expect unidirectional changes, such is for a ChIP-qPCR experiment, where enrichment is assessed for binding a given chromatin region over unbound regions. P values represent * < 0.05; ** < 0.01; *** < 0.001. The number of experiments and biological samples used is specified in figure legends. Error bars represent standard deviation.

DATA AND SOFTWARE AVAILABILITY

The accession number for the RNA-seq, ChIP-seq, and ATAC-seq data reported in this paper is GEO: GSE85178.

Cell Stem Cell, Volume *21*

Supplemental Information

Constitutively Active SMAD2/3 Are Broad-Scope Potentiators of Transcription-Factor-Mediated Cellular Reprogramming

Tyson Ruetz, Ulrich Pfisterer, Bruno Di Stefano, James Ashmore, Meryam Beniazza, Tian V. Tian, Daniel F. Kaemena, Luca Tosti, Wenfang Tan, Jonathan R. Manning, Eleni Chantzoura, Daniella Rylander Ottosson, Samuel Collombet, Anna Johnsson, Erez Cohen, Kosuke Yusa, Sten Linnarsson, Thomas Graf, Malin Parmar, and Keisuke Kaji

Supplemental Figures and Legends

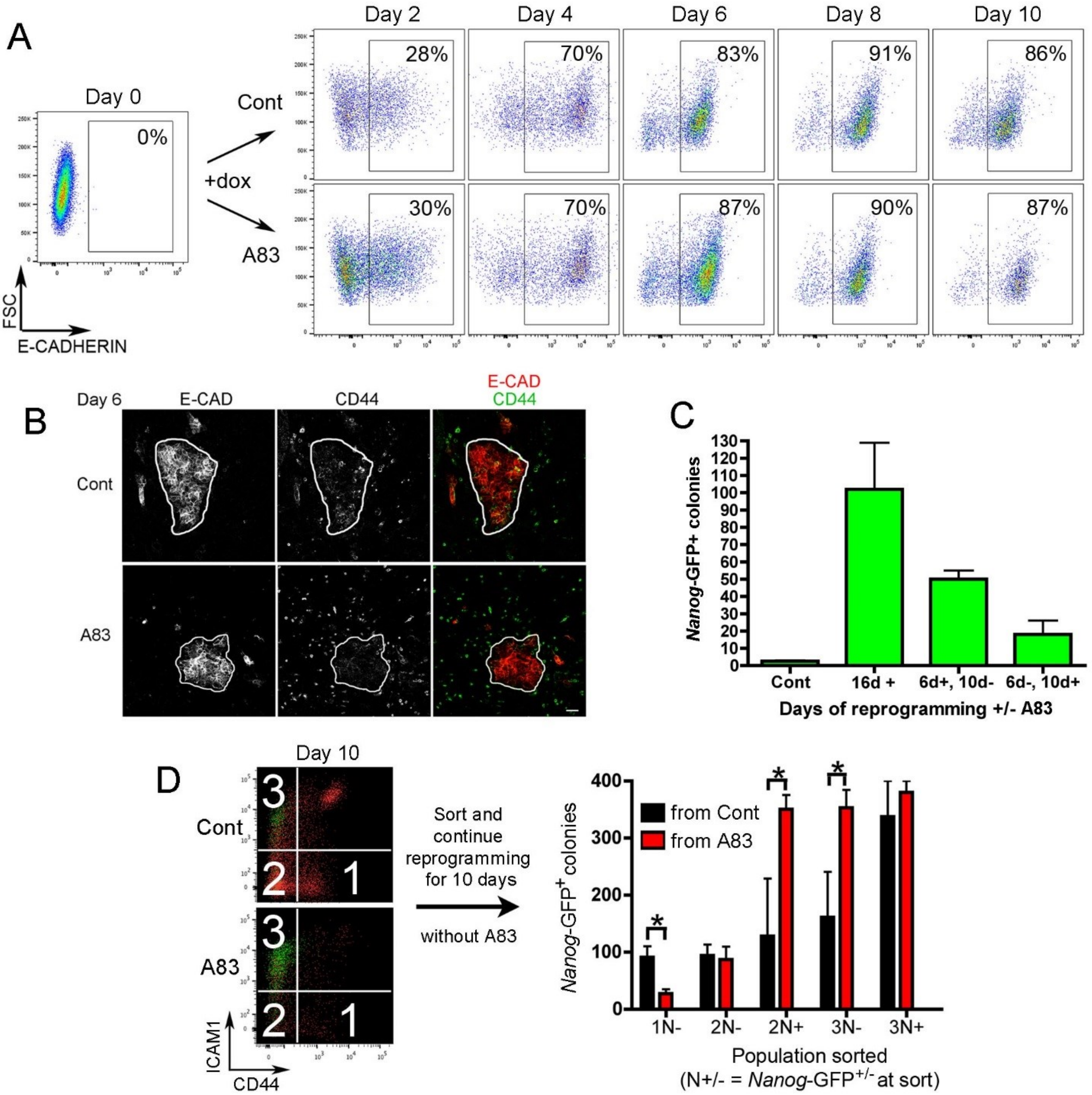

Figure S1, Related to Figure 1. Effects of the TGF-β inhibitor on reprogramming. (A) E-CADHERIN expression changes during reprogramming without (top panels) and with A83 (bottom panels). (B) Confocal microscopy images of E-CAD and CD44 expression at day 6 of reprogramming without (top panels) and with A83 (bottom panels). Scale bar is 50 μM. (C) Numbers of *Nanog*-GFP+ colonies in the absence (Cont) or presence of A83 for 16 days (16d+), only the first 6 days (6d+, 10d-), and the last 10 days (6d-, 10d+). These experiments were performed in the absence of VitC. (D) *Nanog*-GFP$^-$ or GFP$^+$ cells in gate 1, 2, 3 on day 10 of reprogramming without (top) or with A83 (bottom) were sorted. Ten days after continuous reprogramming in the absence of A83, numbers of *Nanog*-GFP$^+$ colonies were counted. Graphs represent averages of 3 independent experiments with 2 technical replicates. Error bars indicate standard deviation (s.d.). *P-value <0.05 based on a one-sided *t*-test.

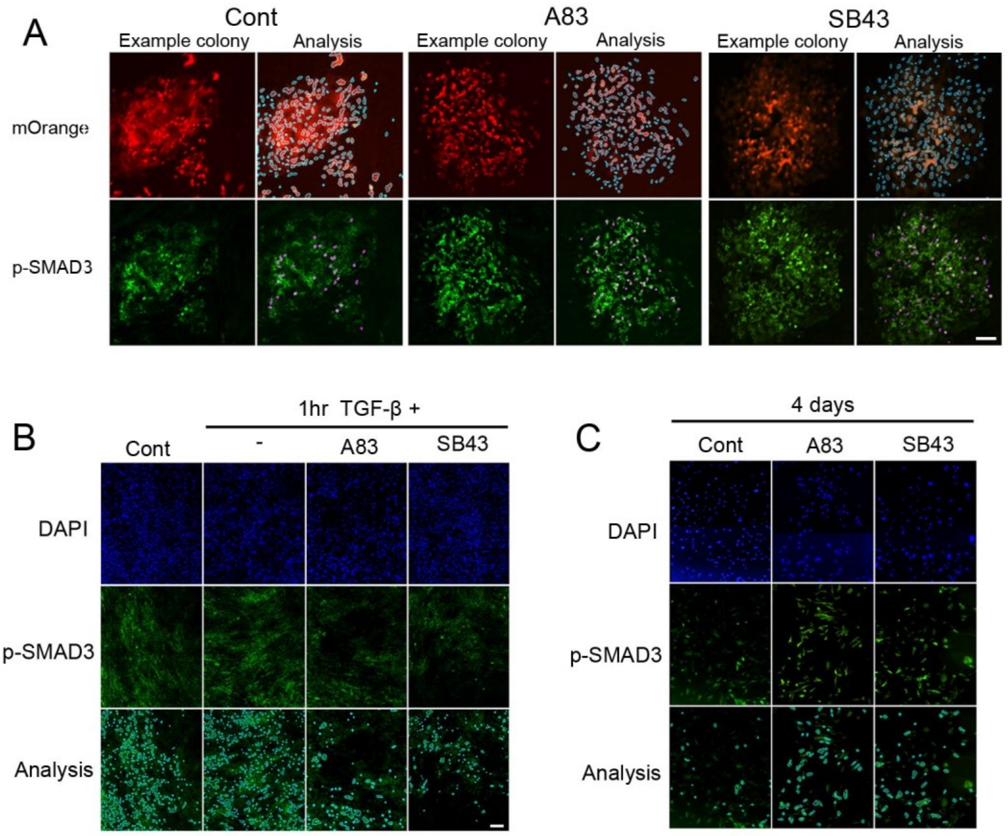

Figure S2, Related to Figure 2. Effects of the TGF-β and SMAD3 inhibitors on p-SMAD3 levels. (A) A close up of whole well images with Celigo imaging cytometer. Cells at day 4 of reprogramming without inhibitors (left), with A83 (middle) or SIS3 (right) were stained for p-SMAD3. Numbers of mOrange$^+$ cells with strong p-SMAD3 foci in each well were quantified with segmentations as indicated. Scale bar is 100 μM. (B, C) Close up images of wild type MEFs cultured for 1 hour (B) or 4 days (C) without inhibitors (Cont, -), with A83 or SB43, stained and quantified for p-SMAD3 as shown. The 1 hour treatments groups were serum starved for 1 hour prior to treatment in ES media. Scale bars are 100 μM.

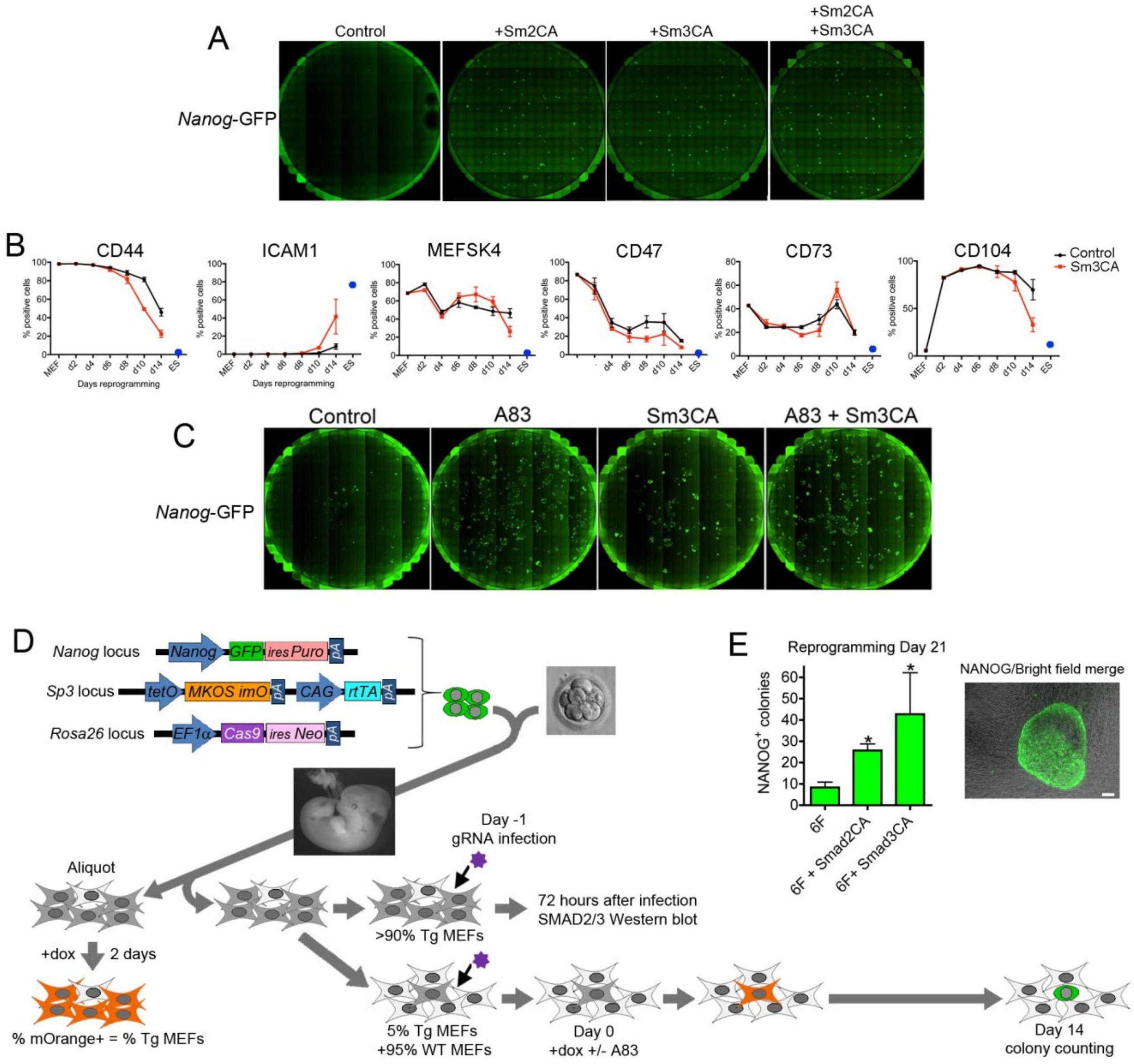

Figure S3, Related to Figure 3. Mouse and human cell reprogramming with Smad2/3CA. (A) Whole well images of reprogramming with control, *Smad2CA*, *Smad3CA*, or *Smad2CA* plus *Smad3CA* expression vector on day 15. Scale bar is 1 cm. (B) Flow cytometry analysis for CD44, ICAM1, MEFSK4, CD47, CD73 and CD104 during reprogramming in the presence (*Sm3CA*) and absence of (Control) of *Smad3CA* expression. (C) Whole well images of reprogramming with control, A83, *Smad3CA*, or A83 plus *Smad3CA* expression vector on day 15. (D) Reprogramming of *Smad2/3* double knockout MEFs. An ESC line with a Nanog-GFP reporter, doxycycline (dox)-inducible *MKOS-ires-mOrange*, CAG promoter-driven *rtTA*, EF1a promoter-driven *Cas9* was used to generate transgenic (Tg) MEFs. A portion of Tg MEFs were used to determine contribution of Tg cells by measuring the % of mOrange$^+$ cells 2 days after dox administration. MEFs from each embryo with >90% Tg cell contribution were pooled and used for further experiments. Infection of gRNA expression viruses was performed onto >90% Tg MEFs and loss of SMAD2/3 protein was assessed 72 hours after infection. A mixture of Tg MEFs and wild-type (WT) MEFs (5 : 95) was infected with the same gRNA expression viruses in parallel and reprogrammed by administration of dox after 24 hours after infection in the presence or absence of A83. (E) Human dermal fibroblast reprogramming using episomal vectors with 6 factors (6F; *OCT3/4*, *SOX2*, *KLF4*, *L-MYC*, *LIN28*, shRNA against P53) with *Smad2CA* or *Smad3CA*. NANOG$^+$ colony numbers were evaluated on day 21 of reprogramming. Graph represents averages of 3 independent experiments, with 2 technical replicates. Error bars indicate standard deviation (s.d.). *P-value <0.05 based on a one-sided *t*-test. Image shows a typical NANOG$^+$ colony in 6F+*Smad3CA* reprogramming. Scale bar is 100 μM.

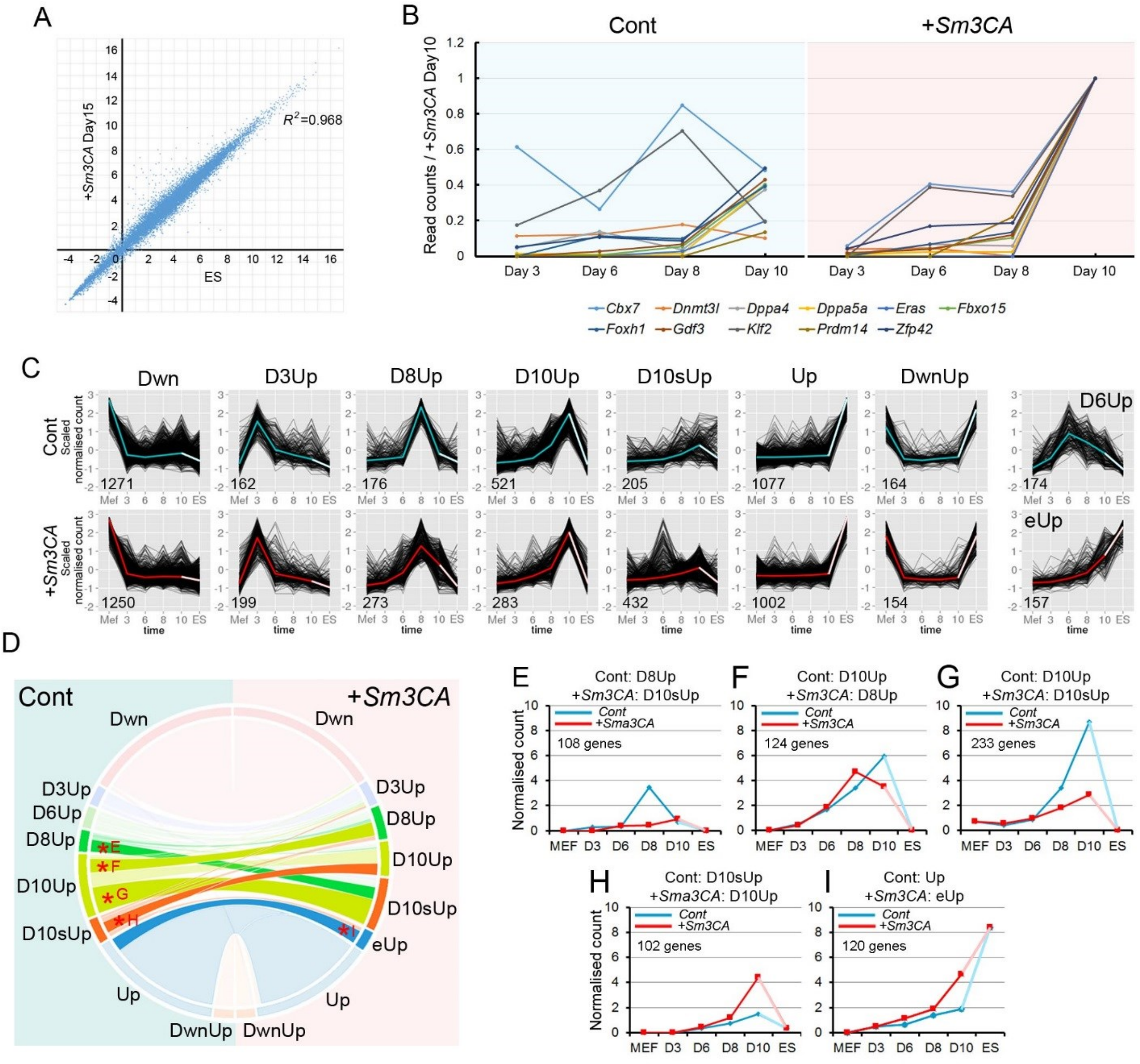

J

	Fold Enrichment Rank	GO biological process complete	REFLIST (22320)	Input (98)	Expected	Fold Enrichment	P-value
E Cont: D8Up +Sm3CA: D10sUp	1	oligodendrocyte differentiation (GO:0048709)	57	5	0.25	19.98	4.69E-02
	2	synapse organization (GO:0050808)	136	7	0.6	11.72	2.06E-02
	3	regulation of synaptic plasticity (GO:0048167)	160	8	0.7	11.39	4.80E-03
	4	regulation of neurotransmitter levels (GO:0001505)	171	8	0.75	10.66	7.85E-03
	5	regulation of synapse structure or activity (GO:0050803)	263	10	1.15	8.66	2.18E-03
	6	modulation of synaptic transmission (GO:0050804)	334	11	1.47	7.5	2.21E-03
	7	synaptic transmission (GO:0007268)	316	10	1.39	7.21	1.13E-02
	8	trans-synaptic signaling (GO:0099537)	316	10	1.39	7.21	1.13E-02
	9	anterograde trans-synaptic signaling (GO:0098916)	316	10	1.39	7.21	1.13E-02
	10	synaptic signaling (GO:0099536)	321	10	1.41	7.1	1.30E-02
F Cont: D10Up Sm3CA: D8Up	1	inflammatory response (GO:0006954)	387	11	1.94	5.66	3.47E-02
	2	innate immune response (GO:0045087)	472	12	2.37	5.07	3.86E-02
	3	immune response (GO:0006955)	861	19	4.32	4.4	4.74E-04
	4	immune system process (GO:0002376)	1646	24	8.26	2.91	1.45E-02
G Cont: D10Up Sm3CA: D10sUp	1	cellular response to interferon-beta (GO:0035458)	30	12	0.27	44.64	1.30E-12
	2	response to interferon-beta (GO:0035456)	36	14	0.32	43.4	5.57E-15
	3	T cell migration (GO:0072678)	11	4	0.1	40.58	2.74E-02
	4	response to interferon-alpha (GO:0035455)	19	5	0.17	29.37	7.65E-03
	5	negative regulation of viral genome replication (GO:0045071)	40	10	0.36	27.9	4.37E-08
	6	defense response to virus (GO:0051607)	127	23	1.14	20.21	5.98E-19
	7	response to interferon-gamma (GO:0034341)	72	13	0.65	20.15	1.60E-09
	8	cellular response to interferon-gamma (GO:0071346)	52	9	0.47	19.32	1.24E-05
	9	regulation of viral genome replication (GO:0045069)	65	10	0.58	17.17	4.62E-06
	10	response to virus (GO:0009615)	166	25	1.49	16.81	6.10E-19

Figure S4, Related to Figure 4. Accelerated gene expression changes toward a pluripotent state by *Smad3CA*. (A) Global gene expression comparison between +*Sm3CA* reprogramming *Nanog*-GFP$^+$ cells on day 15 and ESCs. (B) Pluripotency gene expression changes during reprogramming with control (Cont) or *Smad3CA* (+*Sm3CA*) vector expression. Among 30 pluripotency genes highlighted in Figure 3A, 11 genes with >2-fold expression in the +*Sm3CA* sample compared to the control sample on day 10 are shown. Mean read counts of triplicates were normalized by the values of the +*Sm3CA* Day 10 sample. (C) Scaled expression profiles for genes clustered by similar expression patterns during reprogramming with control (Cont) and *Smad3CA* (+*Sm3CA*) vector expression. The blue and red lines represent mean values of each cluster. Numbers of genes in each category are indicated in each panel. (D) Distribution of genes classified in each category in control (left) and +*Smad3CA* (right) reprogramming. Asterisks indicate cross-classified gene groups with more than 100 genes. (E-I) Un-scaled expression patterns of the cross-classified gene groups *E-I in D. The median values of normalized counts in each group are indicated as blue or red lines from control or +*Smad3CA* reprogramming, respectively. (J) Gene ontology enriched in *E-G groups with PANTHER (Thomas et al., 2003). No enrichment was observed in *H-I groups.

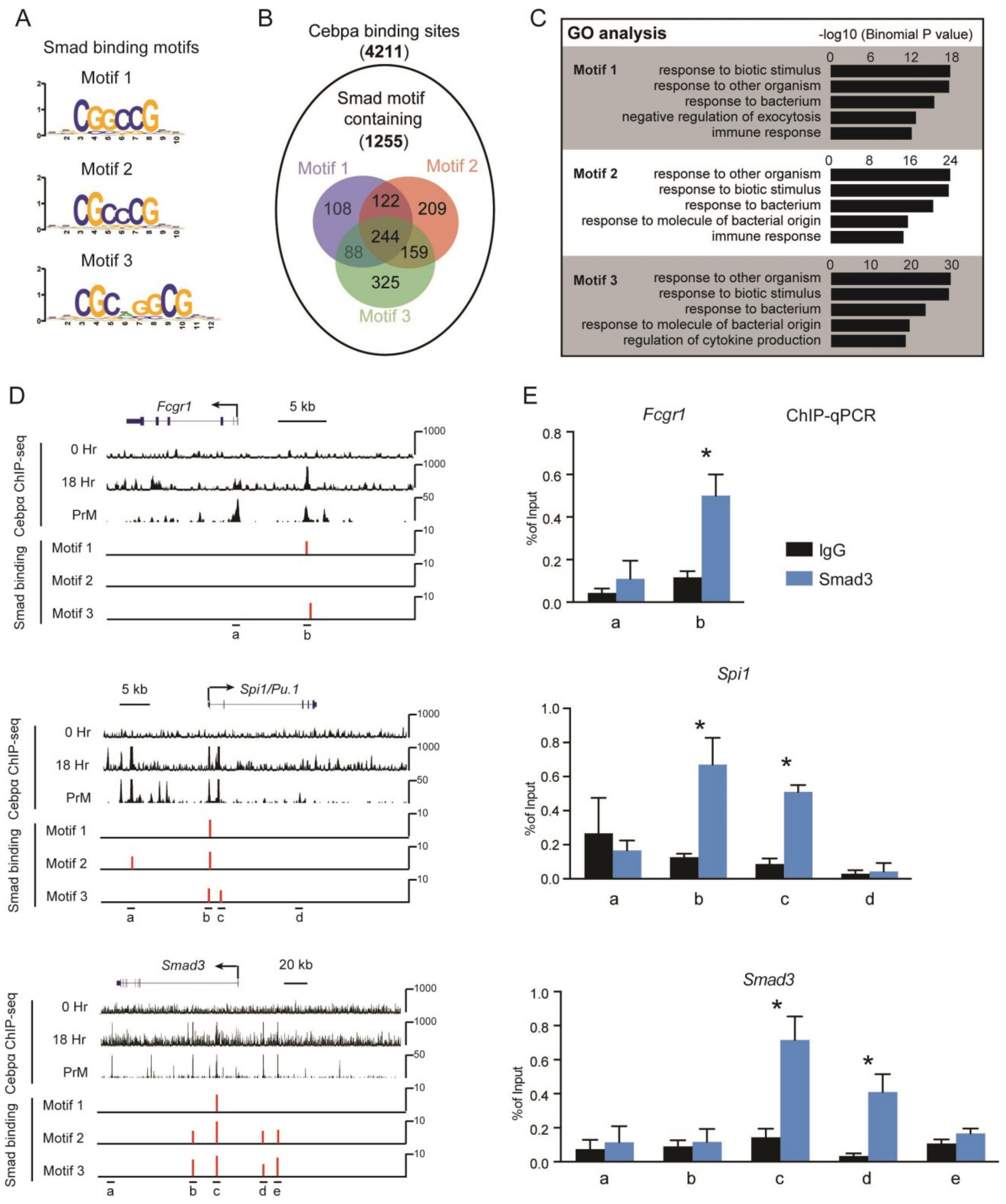

Figure S5, Related to Figure 6. Smad3 binds to Cebpα-bound genomic loci during transdifferentiation from B cells to macrophages. (A) Three Smad-binding motifs found by motif discovery in the vicinity of 4211 Cebpα peak summits. The Cebpα ChIP-seq was performed 18 hours after its induction in primary mouse CD19+ B cells. (GSE71218) (Di Stefano et al., 2016) (B) Venn diagram showing numbers of the three motifs from (A) and the overlap between them. (C) Gene Ontology (GO) enrichments for Cebpα target genes associated with indicated Smad motif from (A). (D) Representative snapshots of loci showing binding of Cebpα (ChIP-seq data in black) and occurrences of Smad-binding motifs (red vertical lines, vertical axes represent the weight of the motif matrix at this position). Cebpα ChIP-seqs were performed in mouse CD19+ B cells (0 Hr) (GSE71218), 18 hours after the induction of Cebpα (18 Hrs) (GSE71218) and in mouse primary macrophages (PrM) (GSE53362) (van Oevelen et al., 2015). (E) Validation of Smad binding by ChIP-qPCR in C10 pre-B cell line induced for Cebpα expression for 18 hrs. S.D. are from two biological replicates (* P<0.05).

A

Intrinsic properties	Cont	+*Sm2CA*	+*Sm3CA*
Resting membrane potential (mV)	-30.14 ± 3.83	-27.11 ± 3.85	-38.20 ± 2.78
Capacitance (pF)	3.98 ± 0.55	7.62 ± 0.70*	8.20 ± 0.69*
Membrane resistance (GΩ)	4.24 ± 1.17	4.40 ± 0.47	4.62 ± 0.93
Inward Na+ current	73.14 ± 43.7	332 ± 46.99*	353.13 ± 52.37*
Outward K+ current	299.28 ± 59.78	665 ± 155.8	827.20 ± 90.04*

* P<0.05

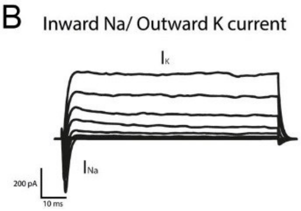

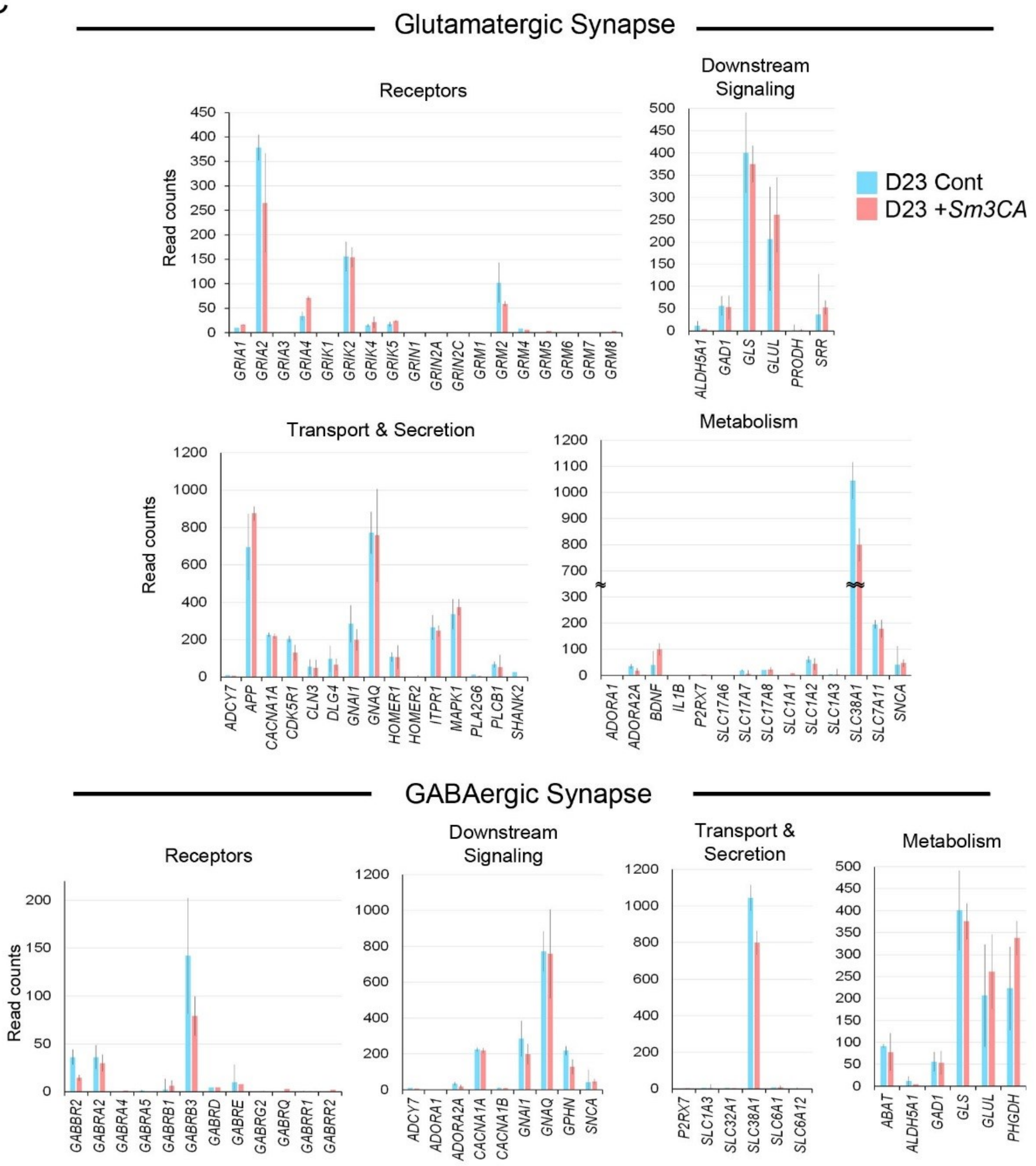

Figure S6, Related to Figure 7. Characters of iNs generated in the presence and absence of Smad3CA. (A) A summary of the main electrophysiological intrinsic membrane parameters measured for hiNs induced with *ABMN* (Cont) or *ABMN* plus *Sm2CA* or *Sm3CA* (25d after conversion). Both membrane capacitance and Na^+, K^+ currents were significantly greater (P<0.05 unpaired t-test) in +*Sm2/3CA* conditions compared to control, indicating a more mature neuronal phenotype. (B) Representative trace of whole-cell currents from hiNs induced with *ABMN* and *Sm3CA*. Trace shows inward sodium (Na^+) and outward potassium (K^+) currents upon depolarization indicative of functional expression of these membrane receptors and a neuronal and mature phenotype. 14 of 15 cells showed the recorded activity. (C) Expression of glutamatergic and GABAergic neuron associated genes. Day 23 *ABMN* (D23 Cont) iNs in pale blue and *ABMN*+*Smad3CA* (D23 +*Sm3CA*) iNs in pink. Mean read counts of triplicates from the RNA-seq are shown. Error bars indicate standard deviation.
